# Relationship between plasma brain-derived neurotrophic factor levels and neurological disorders: An investigation using Mendelian randomisation

**DOI:** 10.1016/j.heliyon.2024.e30415

**Published:** 2024-04-26

**Authors:** Wei Wang, Runshi Gao, Xiaoming Yan, Wei Shu, Xi Zhang, Wenjie Zhang, Lan Zhang

**Affiliations:** a Department of Pharmacy, Xuanwu Hospital, Capital Medical University, Beijing, China; b Department of Functional Neurosurgery, Xuanwu Hospital, Capital Medical University, Beijing, China

**Keywords:** BDNF, Mendelian randomisation, Neurological disorders, Risk factors

## Abstract

**Background:**

Altered brain-derived neurotrophic factor (BDNF) concentrations have been detected in the central nervous system tissues and peripheral blood. These alterations are associated with a series of neurological disorders.

**Objective:**

To investigate the potential causal relationships between genetically determined plasma BDNF levels and various neurological diseases using a two-sample Mendelian randomisation study.

**Methods:**

We selected single nucleotide polymorphisms strongly related to plasma BDNF levels as instrumental variables. Within the Mendelian randomisation framework, we used summary-level statistics for exposure (plasma BDNF levels) and outcomes (neurological disorders).

**Results:**

We observed suggestive evidence of a relation between higher plasma BDNF levels and less risk of nontraumatic intracranial haemorrhage (nITH) (odds ratio [OR] = 0.861, 95 % confidence interval [CI]: 0.774–0.958, *P* = 0.006, *P*_*FDR*_ = 0.078), epilepsy (OR = 0.927, 95 % CI: 0.880–0.976, *P* = 0.004, *P*_*FDR*_ = 0.078), focal epilepsy (OR = 0.928, 95 % CI: 0.874–0.986, *P* = 0.016, *P*_*FDR*_ = 0.139), and non-lesional focal epilepsy (OR = 0.981, 95 % CI: 0.964–0.999, *P* = 0.041, *P*_*FDR*_ = 0.267). Combined with the UK Biobank dataset, the association of plasma BDNF levels with nITH remained significant (OR = 0.88, 95 % CI: 0.81–0.96, *P* < 0.01). The combined analysis of three consortium datasets demonstrated a considerable impact of plasma BDNF on epilepsy (OR = 0.94, 95 % CI: 0.90–0.98, *P* < 0.01) and a suggestive impact on focal epilepsy (OR = 0.94, 95 % CI: 0.89–0.99, *P* = 0.02). However, there was no apparent correlation between plasma BDNF levels and other neurological disorders or related subtypes.

**Conclusions:**

Our study supports a possible causal relationship between elevated plasma BDNF levels and a reduced risk of nITH, epilepsy, and focal epilepsy.

## 1 Introduction

Biomarkers facilitate the differential diagnosis and prognostic assessment of neurological disorders. One such biomarker is the brain-derived neurotrophic factor (BDNF), which belongs to a protein family called neurotrophins that supports central nervous system (CNS) function [1]. BDNF is primarily synthesised in the neuronal and glial cell bodies of the brain, where it is delivered to the pre- and postsynaptic terminals and dendrites [2]. Proneurotrophin (pro-BDNF), a precursor of BDNF, is cleaved within the cytoplasm to produce mature BDNF [3]. Mature BDNF binds to tyrosine kinase receptors (Trks), particularly TrkB, improving long-term potentiation, neuronal survival, and synaptic plasticity. In contrast, pro-BDNF binds to p75 receptors and counteracts the effects of BDNF by causing long-term depression (LTD) and apoptosis [[4], [5], [6]]. Changes in BDNF levels have been observed in both CNS tissues and circulating blood. The pathogenesis of various neurodegenerative disorders, such as Parkinson's disease (PD), Huntington's disease, Alzheimer's disease (AD), multiple sclerosis (MS), ischaemic stroke, and amyotrophic lateral sclerosis (ALS), is associated with these alterations in the body [7]. In addition, a meta-analysis showed that compared to those in healthy individuals, peripheral blood BDNF levels in patients with obsessive-compulsive disorder were significantly reduced, indicating that BDNF may serve as a potential biomarker for obsessive-compulsive disorder [8]. Transplantation of serum exosomes from patients with schizophrenia into normal mice can lead to changes in BDNF gene expression in the hippocampus and prefrontal cortex of mice [9], and hsa-miR-206 in exosomes can regulate BDNF expression [10].

Compared to healthy individuals, patients with AD have differentially upregulated and downregulated BDNF levels [11,12]. Patients with PD also exhibit decreased BDNF concentrations in the substantia nigra and post-mortem brain tissue [13]. Additionally, patients newly diagnosed with PD have significantly lower BDNF levels. In contrast, patients with ALS tend to have higher serum BDNF levels than healthy individuals [14]. In individuals with MS, low BDNF levels are associated with increased interleukin (IL)-6 levels and worse cognitive task scores [15]. Low circulating BDNF levels are a well-established risk factor for stroke and impaired recovery, and stroke acutely stimulates BDNF expression in the brain [16]. Nowroozi et al. conducted a systematic meta-analysis and review and found that individuals with epilepsy generally have similar BDNF levels to those in healthy controls, even though lower BDNF levels are expressed in individuals with partial epilepsy [17]. Tanure et al. observed dramatically higher serum BDNF levels during a migraine attack than during a pain-free period [18]. Although numerous studies have reported altered BDNF levels in the brain and plasma of patients with various brain pathologies, conclusive evidence is still unavailable to determine whether these alterations in BDNF levels result from illness onset. Therefore, establishing the causative role of BDNF in neurological disorders is crucial.

Mendelian randomisation (MR) studies use instrumental variables (IVs), typically single nucleotide polymorphisms (SNPs), to identify the causal effect of exposure on outcomes [19]. When reliable randomised controlled trials (RCTs) are lacking or conducting new RCTs is not feasible, MR can be an effective alternative method for elucidating the causal relationship between exposures and outcomes [20]. MR offers advantages in controlling confounding factors that may introduce bias in observational investigations by leveraging the random allocation of genetic alleles to the offspring before birth [21,22]. Moreover, MR can mitigate reverse causality because genotype formation precedes disorder onset and is unaffected by disorder progression [20]. Herein, we used data from the largest genome-wide association study (GWAS) to date on plasma BDNF levels, along with a statistical summary of seven common neurological disorders (AD, PD, ALS, MS, epilepsy, stroke, and migraine). We conducted a two-sample MR study to investigate the causal relationship between genetic predisposition to plasma BDNF levels and the risk of various neurological illnesses and related subtypes.

## 2 Materials and methods

**Fig. 1:**
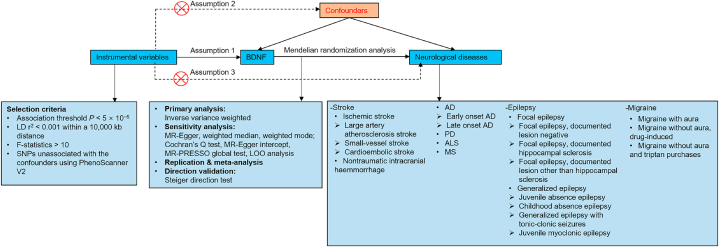
Summary of the present Mendelian randomisation research. Hypothesis 1, genetic variants are strongly and consistently related to exposures being studied; Hypothesis 2, genetic variants are unrelated to any confounding factor capable of influencing the relationship between exposures and outcomes; Hypothesis 3, genetic variants only affect the outcome through exposure. Abbreviations: BDNF, brain-derived neurotrophic factor; LD, linkage disequilibrium; SNPs, single nucleotide polymorphisms; LOO, leave-one-out; AD, Alzheimer's disease; PD, Parkinson's disease; ALS, amyotrophic lateral sclerosis; MS, multiple sclerosis.

An univariable two-sample MR analysis investigated the potential causal associations between plasma BDNF levels and various neurological disorders, including PD, MS, AD, stroke, ALS, migraine, and epilepsy (Table 1). The MR framework used in this study is illustrated in Fig. 1. To analyse the causal impact of exposure on outcomes, three assumptions were considered: first, the genetic variants used should have strong associations with the risk; second, genetic variants should not be confounded by other variables; and third, genetic variants should only influence outcomes through exposure [20]. Additionally, we conducted a reverse MR analysis to rule out the possibility of reverse causality. As we utilised publicly available GWAS summary data, this study required no additional ethical approval.

**Table 1:** Detailed information of utilised studies.

Phenotype	Consortium	Sample size (cases/controls)	Population	Journal	Year	References
Plasma BDNF	–	3301	European	Nature	2018	Sun et al.
Stroke	MEGASTROKE	40,585/406,111	European	Nat Genet	2018	Malik et al.
Ischemic stroke	MEGASTROKE	34,217/406,111	European	Nat Genet	2018	Malik et al.
Large artery atherosclerosis stroke	MEGASTROKE	4373/406,111	European	Nat Genet	2018	Malik et al.
Small-vessel stroke	MEGASTROKE	5386/192,662	European	Nat Genet	2018	Malik et al.
Cardioembolic stroke	MEGASTROKE	7193/406,111	European	Nat Genet	2018	Malik et al.
Nontraumatic intracranial haemmorrhage	FinnGen	6530/342,673	European	–	2023	–
Neurodegenerative disease						
Alzheimer's disease	FinnGen	9301/367,976	European	–	2023	–
Early onset Alzheimer's disease	FinnGen	1314/170,429	European	–	2023	–
Late onset Alzheimer's disease	FinnGen	6489/170,429	European	–	2023	–
Parkinson's disease	IPDGC	33,674/449,056	European	Lancet Neurol	2019	Nalls et al.
Amyotrophic lateral sclerosis	AVS	20,806/59,804	European	Neuron	2018	Nicolas et al.
Multiple sclerosis	FinnGen	2182/373,987	European	–	2023	–
Epilepsy	ILAEC	15,212/29,677	Mixed	Nat Commun	2018	Abou-Khalil et al.
Focal epilepsy	ILAEC	9671/29,677	Mixed	Nat Commun	2018	Abou-Khalil et al.
Focal epilepsy-documented lesion negative	ILAEC	2716/29,677	Mixed	Nat Commun	2018	Abou-Khalil et al.
Focal epilepsy-documented hippocampal sclerosis	ILAEC	803/29,677	Mixed	Nat Commun	2018	Abou-Khalil et al.
Focal epilepsy-documented lesion other than hippocampal sclerosis	ILAEC	3070/29,677	Mixed	Nat Commun	2018	Abou-Khalil et al.
Generalized epilepsy	ILAEC	3769/29,677	Mixed	Nat Commun	2018	Abou-Khalil et al.
Juvenile absence epilepsy	ILAEC	415/29,677	Mixed	Nat Commun	2018	Abou-Khalil et al.
Childhood absence epilepsy	ILAEC	793/29,677	Mixed	Nat Commun	2018	Abou-Khalil et al.
Generalized epilepsy with tonic-clonic seizures	ILAEC	288/29,677	Mixed	Nat Commun	2018	Abou-Khalil et al.
Juvenile myoclonic epilepsy	ILAEC	1181/29,677	Mixed	Nat Commun	2018	Abou-Khalil et al.
Migraine	FinnGen	18,477/287,837	European	–	2023	–
Migraine with aura	FinnGen	7917/287,837	European	–	2023	–
Migraine without aura, drug-induced	FinnGen	321/376,956	European	–	2023	–
Migraine without aura and triptan purchases	FinnGen	4989/337,890	European	–	2023	–

Abbreviations: BDNF, brain-derived neurotrophic factor; IPDGC, International Parkinson's Disease Genomics Consortium; AVS, ALS Variant Server; ILAEC, International League Against Epilepsy Consortium.

### 2.1 GWAS of plasma BDNF

Genetic instruments for plasma BDNF in the MR analysis were acquired from a large-scale GWAS. The study included 3301 individuals of European descent who participated in an INTERVAL study [23] containing approximately 50,000 blood donors who were ≥18. Plasma BDNF levels were determined using an aptamer-based multiplexing method known as the SOMA scan assay. Subsequent experiments were conducted to ensure accuracy and reliability of the measurements.

### 2.2 GWAS of neurological disorders

The MEGASTROKE consortium provided GWAS data on stroke, including data from 40,585 patients and 406,111 controls of European descent. The MEGASTROKE consortium also supplied summary-level data on ischaemic stroke for 440,328 individuals (34,217 ischaemic stroke cases and 406,111 controls) and subcategorised them according to the stroke type: large artery atherosclerosis stroke (n = 4373), small-vessel stroke (n = 5386), and cardioembolic stroke (n = 7193) [24]. Additionally, the FinnGen consortium contributed summary-level data to assess the genetic predisposition to non-traumatic intracranial haemorrhage (nITH), which consisted of 6530 cases and 342,673 controls of European descent. According to the definition of the endpoint provided by FinnGen, nITH can be considered synonymous with haemorrhagic stroke [25].

This study utilised genetic variants collected from the International Parkinson's Disease Genomics Consortium (IPDGC) and the ALS variant server (AVS). The IPDGC provided data from 33,674 PD cases and 449,056 control individuals, while the AVS contributed data from 20,806 ALS cases and 59,804 healthy controls [26,27]. The FinnGen consortium supplied summary GWAS data for AD with 9301 AD cases and 367,976 controls. Additionally, the FinnGen consortium provided data for GWAS of AD subtypes. For early-onset AD, 314 individuals with AD and 67,781 controls were included, and for late-onset AD, 1232 individuals with AD and 67,778 controls were studied. Furthermore, the FinnGen consortium provided data for GWAS of MS, including 2182 MS cases and 373,987 controls. It is worth noting that all individuals in the datasets were of European ancestry.

The International League Against Epilepsy Consortium (ILAEC) provided summary data on epilepsy and its subtypes, including 15,212 patients with epilepsy, 9671 individuals with focal epilepsy, 3769 individuals with generalized epilepsy, and 29,677 healthy controls. Additionally, data for seven sub-phenotypes of epilepsy were available: focal epilepsy (documented lesion negative), focal epilepsy (documented hippocampal sclerosis), focal epilepsy (documented lesions other than hippocampal sclerosis), juvenile absence epilepsy, childhood absence epilepsy, generalized epilepsy with tonic-clonic seizures, and juvenile myoclonic epilepsy [28]. The FinnGen consortium provided summary statistical data for migraine together with its subtypes, including 18,477 patients and 287,837 controls of European descent for migraine; 7917 patients and 287,837 controls of European descent for migraine with aura; 321 patients and 376,956 controls of European descent for migraine without aura, drug-induced, together with 4989 patients and 337,890 controls of European descent for migraine without aura and triptan purchases.

Furthermore, in the analysis of nITH, the study utilised data from the UK Biobank (UKB), which consisted of 2437 cases and 407,633 controls of European ancestry. Additionally, this MR study incorporated two separate sets of summary statistics for epilepsy and focal epilepsy obtained from both the FinnGen consortium and the UKB. Summary statistics from the FinnGen consortium included 11,740 cases of epilepsy and 287,837 controls of European descent. Moreover, it encompassed 6213 cases of focal epilepsy and 365,534 controls of European descent. Similarly, statistical summaries from the UKB included 1128 individuals with epilepsy and 403,249 controls of European descent. Additionally, it contained 345 cases of focal epilepsy and 403,249 controls of European descent.

### 2.3 Selection of instruments

Table 1 presents the number of valid instruments (SNPs) for each exposure-outcome pair considered in the MR analysis. Initially, we set p < 5 × 10^−8^ as the genome-wide significant threshold to select SNPs strongly associated with plasma BDNF, but only a limited number of SNPs were identified. Subsequently, we extracted significant SNPs highly correlated with the exposure at a genome-wide significance threshold (P < 5 × 10^−6^) for traits related to plasma BDNF. This process aimed to identify vital candidate instruments. Dependent SNPs with high linkage disequilibrium were excluded from the candidate instrument set using a window size of 10,000 kb and parameter r^2^ > 0.001. We also excluded weak instruments based on their F-statistic, with a threshold of F-statistics <10 [29], and F statistics = (β/SE)^2^. SNPs associated with the outcome at a *P* value < 5 × 10^−5^ were excluded from the instrument set before MR analysis. Additionally, we utilised the PhenoScanner database (Version 2, http://www. phenoscanner. medschl. cam. ac. uk/) [30] to identify other genome-wide significant traits (*P* < 5 × 10^−5^) related to the selected SNPs potentially influencing the included neurological disorders. Related SNPs were then excluded from the analysis (Table S3). The harmonised SNPs for each exposure-outcome combination were documented and stored (Table S4). Palindromic SNPs with middle allele frequencies (MAF) were excluded. SNPs with the A/T or G/C allele and a MAF ranging from 0.01 to 0.30 are categorized as palindromic SNP.

### 2.4 Statistical analysis

We utilised various methods to assess the causal relationship between plasma BDNF levels and neurological disorders. Inverse-variance weighted (IVW) was the default approach, as described in previous studies [31]. Additionally, we conducted sensitivity analyses using the MR-Egger, weighted mode, and weighted median methods to validate the estimates [32]. Detailed explanations of these MR methods can be found in a previous study [33]. To evaluate the heterogeneity of the IVW analysis, we performed Cochran's Q statistics [34]. To detect horizontal pleiotropy, we conducted the MR-pleiotropy residual sum and outlier (MR-PRESSO) global test and the MR-Egger intercept test and visually inspected the funnel plot [35]. Furthermore, a leave-one-out analysis was conducted to survey the impact of individual SNPs on the overall causal implications. Plasma BDNF levels can be considered causally associated with neurological disorders if the following criteria are met: 1) considerable difference in the IVW method (*P* < 0.05); 2) consistent estimation directions among the IVW, MR-Egger, weighted median, and weighted mode methods; and 3) non-significant results in the MR-Egger intercept test and the MR-PRESSO global test (*P* > 0.05) [36]. To account for multiple tests, *P* values corrected by the false discovery rate (FDR) were calculated using the Benjamini–Hochberg approach in MR associative analyses [37]. An association with an FDR-adjusted *P* value below 0.05 was considered significant, whereas an association with a raw *P* value below 0.05 but a *P*_*FDR*_ above 0.05 was considered suggestive evidence.

If the initial *P* value obtained from the IVW analysis was less than 0.05, we performed additional meta-analyses using data from other consortiums to compute the overall causal estimates. In cases with significant heterogeneity (I^2^ < 50 %), a fixed-effects model was used to estimate the combination. Otherwise, a random effects model was employed [38]. Following the approach described by Schmidt et al., we conducted independent Bonferroni corrections for each outcome, which were assessed to account for type I and II errors [39]. A *P* value of 0.05/n (where n denotes the number of GWAS datasets involved) after the Bonferroni correction was considered statistically significant for the GWAS datasets included in this study. Additionally, *P* < 0.05 was considered suggestive of a potential causal relation.

Furthermore, we assessed whether the observed causal relationships were influenced by reverse causation using the Steiger test [40]. This test evaluated whether the included SNPs accounted for a more significant proportion of the variability in neurological disorders than the identified plasma BDNF levels. If combinations of SNPs contributed more to the genetic risk of neurological disorders than to plasma BDNF levels (Steiger *P* > 0.05), a potential bias in the direction of causal inference was suggested. All analyses were performed using TwoSampleMR (version 0.5.7), Mendelian Randomisation (version 0.8.0), and the MRPRESSO package (1.0) in R Software 4.3.1 (https://www.R-project.org).

## 3 Results

### 3.1 Selecting genetic variants

The MR analysis included a selection of SNPs based on specific criteria. The number of SNPs retained is shown in Table 2. The F-statistics for these SNPs were greater than 10, implying the absence of weak instruments (Table S4). The harmonised data used in the analysis are listed in Table S4.

**Table 2:** Major findings of Mendelian randomisation analyses.

Outcome	nSNPs	Method	Beta	OR (95 % CI)	*P* value	*P*_*FDR*_
Stroke	10	IVW	0.027	1.027 (0.972–1.085)	0.343	0.892
Ischemic stroke	10	IVW	0.020	1.020 (0.961–1.083)	0.514	0.94
Large artery atherosclerosis stroke	10	IVW	0.077	1.080 (0.928–1.258)	0.320	0.892
Small-vessel stroke	10	IVW	−0.001	0.999 (0.846–1.179)	0.986	0.986
Cardioembolic stroke	10	IVW	−0.008	0.992 (0.873–1.128)	0.904	0.94
Nontraumatic intracranial haemmorrhage	9	IVW	−0.15	0.861 (0.774–0.958)	**0.006**	0.078
Neurodegenerative disease						
Alzheimer's disease	9	IVW	0.008	1.008 (0.917–1.108)	0.862	0.94
Early onset Alzheimer's disease	9	IVW	−0.080	0.923 (0.726–1.173)	0.511	0.94
Late onset Alzheimer's disease	9	IVW	0.028	1.028 (0.911–1.161)	0.652	0.94
Parkinson's disease	9	IVW	0.094	1.098 (0.973–1.239)	0.130	0.483
Amyotrophic lateral sclerosis	11	IVW	−0.018	0.982 (0.898–1.073)	0.690	0.94
Multiple sclerosis	10	IVW	−0.026	0.974 (0.822–1.154)	0.760	0.94
Epilepsy	6	IVW	−0.076	0.927 (0.880–0.976)	**0.004**	0.078
Focal epilepsy	6	IVW	−0.074	0.928 (0.874–0.986)	**0.016**	0.139
Focal epilepsy-documented lesion negative	6	IVW	−0.004	0.981 (0.964–0.999)	**0.041**	0.267
Focal epilepsy-documented hippocampal sclerosis	6	IVW	−0.002	0.998 (0.986–1.011)	0.795	0.94
Focal epilepsy-documented lesion other than hippocampal sclerosis	6	IVW	−0.014	0.986 (0.968–1.004)	0.123	0.483
Generalized epilepsy	6	IVW	−0.071	0.932 (0.815–1.066)	0.304	0.892
Juvenile absence epilepsy	6	IVW	−0.002	0.998 (0.990–1.006)	0.610	0.94
Childhood absence epilepsy	6	IVW	0.0045	1.005 (0.994–1.015)	0.418	0.906
Generalized epilepsy with tonic-clonic seizures	6	IVW	−0.007	0.993 (0.985–1.002)	0.122	0.483
Juvenile myoclonic epilepsy	6	IVW	−0.007	0.993 (0.976–1.010)	0.397	0.906
Migraine	10	IVW	0.008	1.008 (0.948–1.071)	0.799	0.94
Migraine with aura	10	IVW	0.011	1.011 (0.907–1.126)	0.847	0.94
Migraine without aura, drug-induced	10	IVW	0.030	1.031 (0.664–1.600)	0.894	0.94
Migraine without aura and triptan purchases	10	IVW	−0.014	0.986 (0.880–1.106)	0.816	0.94

The investigation of non-traumatic intracranial haemorrhage utilised FinnGen consortium data while analysing epilepsy, and its subtypes relied on data from the ILAEC dataset.

The presence of bold symbols indicate statistical significance with a threshold of *P* < 0.05.

Abbreviations: SNP, single-nucleotide polymorphism; OR, odds ratio; CI, confidence interval; FDR, false discovery rate; IVW, inverse-variance weighted; ILAEC, International League Against Epilepsy Consortium.

### 3.2 Causal relationship between plasma BDNF and nITH, epilepsy, focal epilepsy, and non-lesional focal epilepsy

**Fig. 2:**
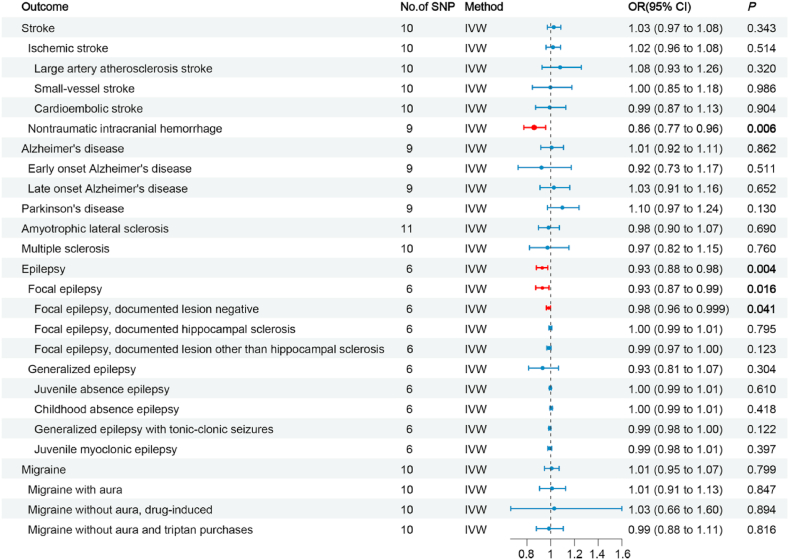
Associations of genetically determined plasma BDNF levels with neurological disorders. Abbreviations: SNP, single nucleotide polymorphism; OR, odds ratio; CI, confidence interval; IVW, inverse-variance weighted.

**Fig. 3:**
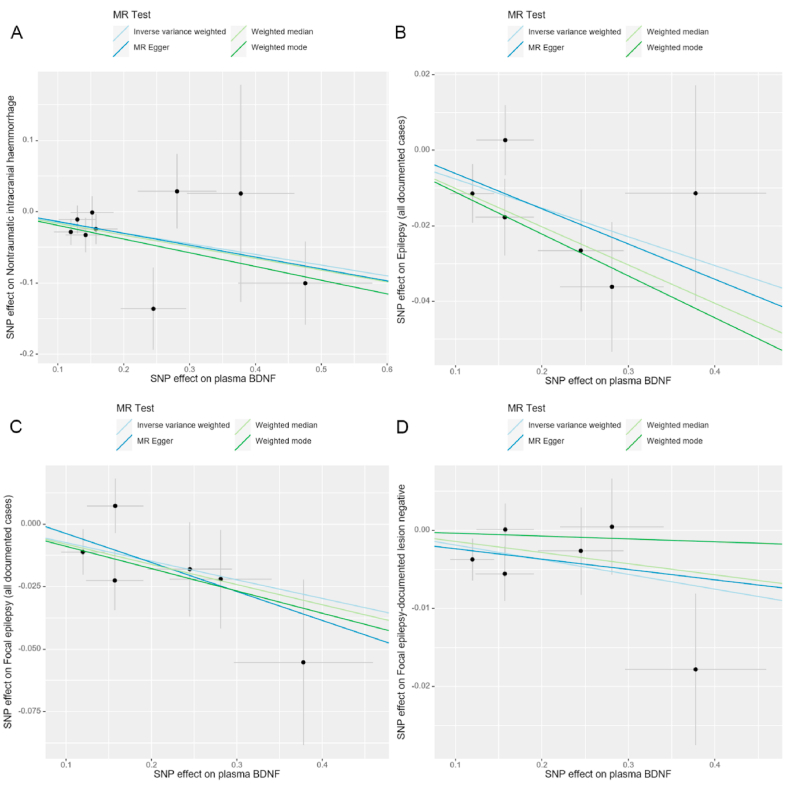
Scatter plots for causal relations of plasma BDNF with **(A)** Nontraumatic intracranial haemorrhage; **(B)** Epilepsy; **(C)** Focal epilepsy; **(D)** Focal epilepsy-documented lesion negative. A straight line's slope demonstrates causal relation magnitude. Abbreviations: BDNF, brain-derived neurotrophic factor.

**Fig. 4:**
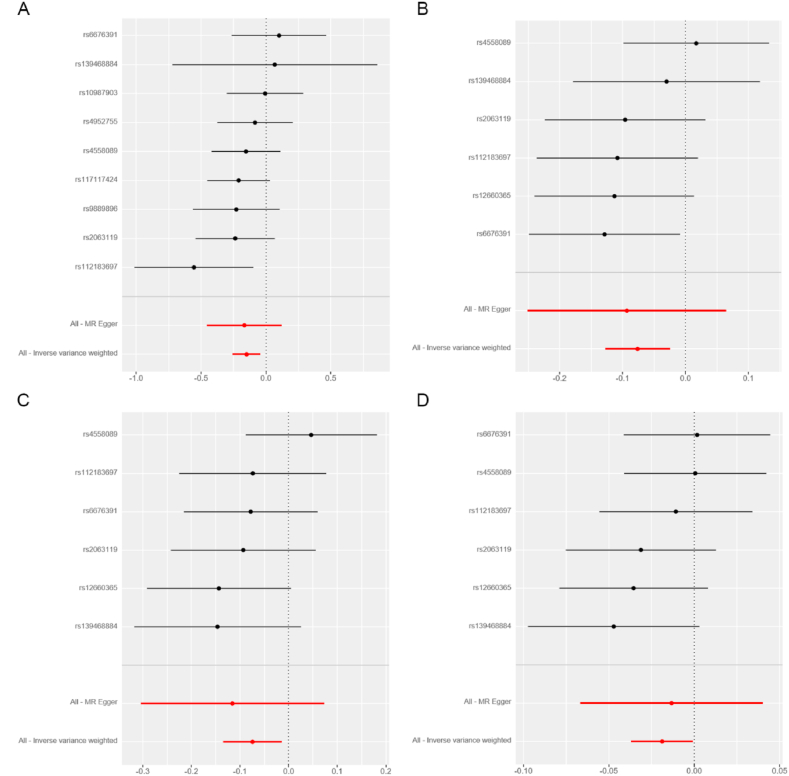
Forest plots for the causal relations of plasma BDNF with **(A)** Nontraumatic intracranial haemorrhage; **(B)** Epilepsy; **(C)** Focal epilepsy; **(D)** Focal epilepsy-documented lesion negative. The horizontal axis displays the effect size of plasma BDNF on each trait in the MR analysis. On the vertical axis, analysis results of all SNPs are depicted. Abbreviations: BDNF, brain-derived neurotrophic factor; MR, Mendelian randomisation; SNP, single nucleotide polymorphism.

**Fig. 5:**
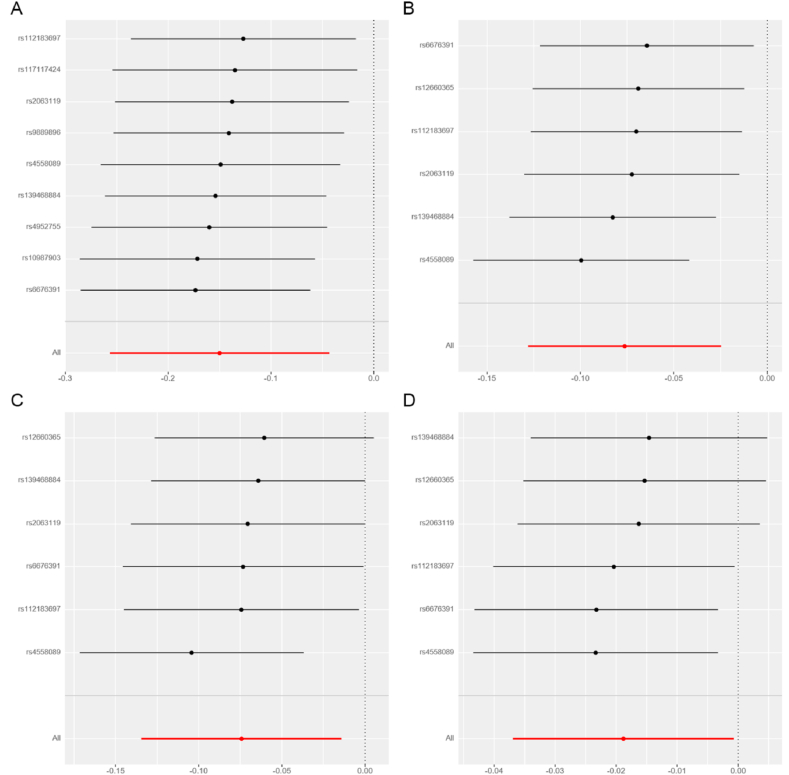
Leave-one-out plots for causal relations of plasma BDNF with **(A)** Nontraumatic intracranial haemorrhage; **(B)** Epilepsy; **(C)** Focal epilepsy; **(D)** Focal epilepsy-documented lesion negative. Abbreviations: BDNF, brain-derived neurotrophic factor.

**Fig. 6:**
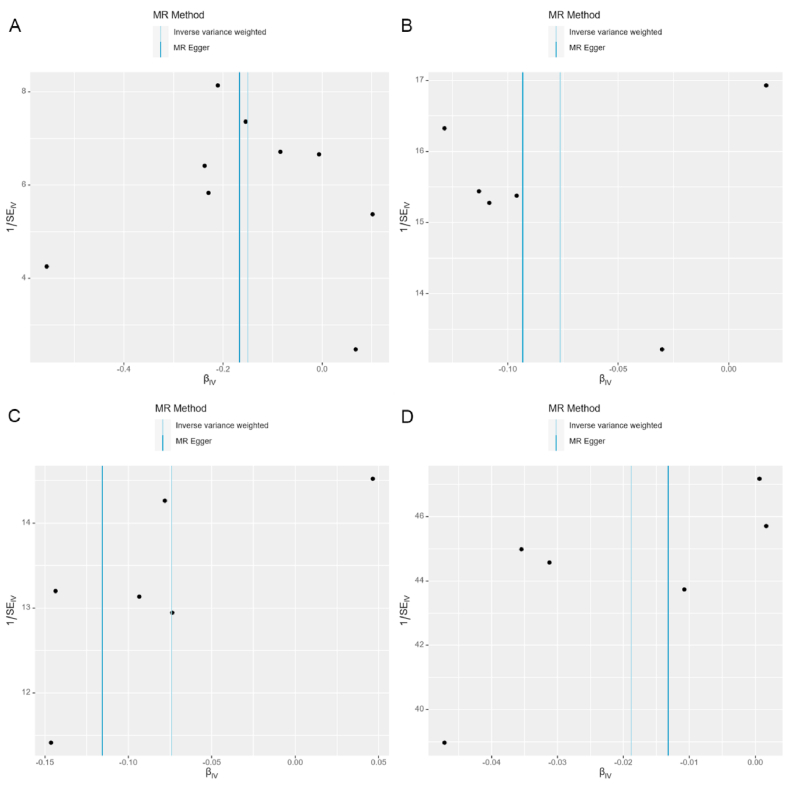
Funnel plots for causal relations of plasma BDNF with **(A)** Nontraumatic intracranial haemorrhage; **(B)** Epilepsy; **(C)** Focal epilepsy; **(D)** Focal epilepsy-documented lesion negative. Abbreviations: BDNF, brain-derived neurotrophic factor.

Based on the results presented in Fig. 2 and Table 2, our analysis using the IVW model suggested that each increased standard deviation (SD) in plasma BDNF decreased the risk of nITH (odds ratio [OR] = 0.861, 95 % confidence interval [CI]: 0.774–0.958, *P* = 0.006, *P*_*FDR*_ = 0.078), epilepsy (OR = 0.927, 95 % CI: 0.880–0.976, *P* = 0.004, *P*_*FDR*_ = 0.078), focal epilepsy (OR = 0.928, 95 % CI: 0.874–0.986, *P* = 0.016, *P*_*FDR*_ = 0.139), and non-lesional focal epilepsy (OR = 0.981, 95 % CI: 0.964–0.999, *P* = 0.041, *P*_*FDR*_ = 0.267). Sensitivity analyses conducted in this study demonstrated that the weighted median estimator yielded statistically significant findings, indicating a significant association between the upregulation of plasma BDNF (per SD) and decreased nITH risk (OR = 0.849; 95 % CI: 0.739–0.975, *P* = 0.021; Table 3). In the sensitivity analyses, the weighted median estimator revealed statistically significant evidence linking plasma BDNF levels (per SD increase) with decreased epilepsy risk (OR = 0.904; 95 % CI: 0.847–0.964, *P* = 0.002; Table 3). Furthermore, the weighted median estimator specifically supported the hypothesis that plasma BDNF levels modestly reduced the risk of focal epilepsy (OR = 0.923; 95 % CI: 0.852–0.998, *P* = 0.046; Table 3). We observed no indications of directional pleiotropy in the analysis of nITH, epilepsy, or various subtypes of epilepsy (all *P*_intercept_ > 0.05; Table 4). It is essential to highlight that all the *P*_*Q*_s are more remarkable than 0.05, except for generalized epilepsy, which was 0.04. Leave-one-out analyses were performed for nITH, epilepsy, focal epilepsy, and non-lesional focal epilepsy. However, none of the SNPs significantly affected the IVW estimate (Fig. 5(A-D)). Applying the Steiger test to the reverse analysis of nITH, epilepsy, and various subtypes of epilepsy indicated potential absence of the outcomes’ causal influence on plasma BDNF (Table S2). The power analysis results are presented in Table S1. Furthermore, Fig. 3(A-D), Fig. 4(A-D), Fig. 5(A-D) and Fig. 6(A-D) depict the scatter, forest, leave-one-out, and funnel plots for nITH, epilepsy, focal epilepsy, and non-lesional focal epilepsy.

**Table 3:** Sensitivity analyses of relations of plasma BDNF levels with neurological disorders.

Outcome	MR-Egger			Weighted median			Weighted mode		
	Beta	OR (95%CI)	*P* value	Beta	OR (95%CI)	*P* value	Beta	OR (95%CI)	*P* value
Stroke	0.065	1.067 (0.911, 1.250)	0.446	0.019	1.019 (0.949, 1.094)	0.597	0.015	1.015 (0.910, 1.133)	0.793
Ischemic stroke	0.009	1.009 (0.850, 1.196)	0.924	0.021	1.021 (0.949, 1.099)	0.570	0.025	1.025 (0.912, 1.153)	0.685
Large artery atherosclerosis stroke	−0.017	0.983 (0.632, 1.531)	0.942	0.029	1.029 (0.830, 1.276)	0.791	−0.056	0.946 (0.676, 1.324)	0.752
Small-vessel stroke	−0.138	0.871 (0.533, 1.424)	0.598	0.082	1.085 (0.888, 1.327)	0.425	0.153	1.166 (0.863, 1.574)	0.344
Cardioembolic stroke	0.287	1.333 (0.942, 1.886)	0.144	−0.022	0.978 (0.831, 1.151)	0.790	−0.072	0.931 (0.735, 1.179)	0.567
Nontraumatic intracranial haemmorrhage	−0.167	0.847 (0.634, 1.130)	0.295	−0.164	0.849 (0.739, 0.975)	**0.021**	−0.192	0.825 (0.691, 0.986)	0.068
Neurodegenerative diseases									
Alzheimer's disease	0.095	1.099 (0.852, 1.419)	0.490	0.015	1.015 (0.895, 1.152)	0.814	0.019	1.019 (0.871, 1.193)	0.817
Early onset Alzheimer's disease	−0.220	0.802 (0.419, 1.538)	0.528	−0.094	0.910 (0.671, 1.236)	0.547	−0.099	0.906 (0.589, 1.393)	0.664
Late onset Alzheimer's disease	0.092	1.096 (0.789, 1.523)	0.600	0.020	1.020 (0.874, 1.191)	0.801	0.021	1.021 (0.839, 1.241)	0.842
Parkinson's disease	0.283	1.327 (0.978, 1.800)	0.112	0.075	1.078 (0.918, 1.266)	0.362	0.074	1.077 (0.865, 1.339)	0.526
Amyotrophic lateral sclerosis	0.105	1.111 (0.860, 1.435)	0.441	−0.049	0.952 (0.858, 1.056)	0.349	−0.047	0.954 (0.844, 1.077)	0.464
Multiple sclerosis	−0.128	0.880 (0.538, 1.439)	0.624	−0.003	0.997 (0.806, 1.233)	0.978	−0.091	0.913 (0.672, 1.242)	0.577
Epilepsy	−0.093	0.911 (0.778, 1.067)	0.312	−0.101	0.904 (0.847, 0.964)	**0.002**	−0.111	0.895 (0.798, 1.004)	0.116
Focal epilepsy	−0.116	0.891 (0.738, 1.075)	0.295	−0.081	0.923 (0.852, 0.998)	**0.046**	−0.089	0.915 (0.809, 1.034)	0.213
Focal epilepsy-documented lesion negative	−0.013	0.987 (0.935, 1.041)	0.654	−0.014	0.986 (0.963, 1.009)	0.234	−0.004	0.996 (0.962, 1.031)	0.844
Focal epilepsy-documented hippocampal sclerosis	−0.014	0.986 (0.948, 1.027)	0.542	0.001	1.001 (0.987, 1.014)	0.903	0.003	1.003 (0.983, 1.023)	0.787
Focal epilepsy-documented lesion other than hippocampal sclerosis	−0.008	0.992 (0.939, 1.047)	0.782	−0.020	0.980 (0.957, 1.003)	0.093	−0.024	0.977 (0.945, 1.009)	0.216
Generalized epilepsy	−0.023	0.978 (0.626, 1.528)	0.926	−0.066	0.936 (0.828, 1.058)	0.290	−0.125	0.883 (0.723, 1.078)	0.276
Juvenile absence epilepsy	0.003	1.003 (0.978, 1.029)	0.844	−0.003	0.997 (0.987, 1.007)	0.535	−0.005	0.995 (0.980, 1.010)	0.558
Childhood absence epilepsy	0.0049	1.005 (0.973, 1.038)	0.783	0.0001	1.000 (0.987, 1.014)	0.988	−0.0018	0.998 (0.980, 1.017)	0.855
Generalized epilepsy with tonic-clonic seizures	−0.015	0.985 (0.959, 1.012)	0.348	−0.008	0.992 (0.984, 1.001)	0.082	−0.011	0.989 (0.977, 1.002)	0.159
Juvenile myoclonic epilepsy	0.005	1.005 (0.952, 1.061)	0.870	−0.008	0.992 (0.975, 1.009)	0.363	−0.007	0.993 (0.970, 1.015)	0.543
Migraine	0.029	1.029 (0.862, 1.230)	0.759	−0.002	0.998 (0.922, 1.081)	0.965	0.022	1.022 (0.917, 1.140)	0.699
Migraine with aura	−0.056	0.945 (0.680, 1.315)	0.748	0.009	1.009 (0.880, 1.156)	0.900	−0.137	0.872 (0.687, 1.107)	0.290
Migraine without aura, drug-induced	−0.991	0.371 (0.102, 1.344)	0.170	0.218	1.243 (0.703, 2.200)	0.454	0.299	1.349 (0.513, 3.542)	0.559
Migraine without aura and triptan purchases	−0.045	0.956 (0.685, 1.334)	0.799	−0.085	0.919 (0.791, 1.068)	0.270	−0.112	0.894 (0.708, 1.129)	0.371

The investigation of non-traumatic intracranial haemorrhage utilised the FinnGen consortium data, while the analysis of epilepsy, together with its subtypes, relied on data from the ILAEC dataset.

The presence of bold symbols indicate statistical significance with a threshold of *P* < 0.05.

Abbreviations: BDNF, brain-derived neurotrophic factor; MR, Mendelian randomisation; OR, odds ratio; CI, confidence interval; ILAEC, International League against Epilepsy Consortium.

**Table 4:** Heterogeneity and pleiotropy tests for relations between plasma BDNF levels and neurological disorders.

Outcome	Cochrane's Q test		MR-Egger Intercept test		MR-PRESSO global test
	Q-value	*P*_*Q*_	Intercept	*P*_intercept_	*P* value
Stroke	3.148	0.958	−0.007	0.628	0.957
Ischemic stroke	1.756	0.995	0.002	0.891	0.996
Large artery atherosclerosis stroke	7.445	0.591	0.017	0.668	0.597
Small-vessel stroke	12.589	0.182	0.024	0.578	0.202
Cardioembolic stroke	10.501	0.311	−0.052	0.114	0.315
Nontraumatic intracranial haemmorrhage	6.978	0.539	0.003	0.906	0.603
Neurodegenerative diseases					
Alzheimer's disease	4.551	0.804	−0.015	0.498	0.822
Early onset Alzheimer's disease	3.387	0.908	0.025	0.664	0.912
Late onset Alzheimer's disease	2.228	0.973	−0.011	0.693	0.981
Parkinson's disease	4.228	0.836	−0.038	0.226	0.858
Amyotrophic lateral sclerosis	12.708	0.240	−0.022	0.340	0.267
Multiple sclerosis	6.300	0.710	0.018	0.678	0.734
Epilepsy	4.246	0.515	0.003	0.834	0.544
Focal epilepsy	4.656	0.459	0.008	0.671	0.502
Focal epilepsy-documented lesion negative	3.931	0.559	−0.001	0.838	0.595
Focal epilepsy-documented hippocampal sclerosis	7.712	0.173	0.002	0.568	0.210
Focal epilepsy-documented lesion other than hippocampal sclerosis	1.999	0.849	−0.001	0.824	0.855
Generalized epilepsy	11.642	**0.040**	−0.009	0.834	0.066
Juvenile absence epilepsy	4.555	0.473	−0.001	0.712	0.509
Childhood absence epilepsy	3.932	0.559	−6.9 × 10^−5^	0.982	0.563
Generalized epilepsy with tonic-clonic seizures	9.713	0.084	0.001	0.575	0.124
Juvenile myoclonic epilepsy	8.299	0.141	−0.002	0.668	0.187
Migraine	3.149	0.958	−0.004	0.812	0.954
Migraine with aura	12.684	0.177	0.012	0.685	0.182
Migraine without aura, drug-induced	7.935	0.541	0.178	0.136	0.539
Migraine without aura and triptan purchases	5.904	0.749	0.005	0.851	0.741

The investigation of non-traumatic intracranial haemorrhage utilised the FinnGen consortium data, while the analysis of epilepsy, together with its subtypes, relied on data from the ILAEC dataset.

Bold symbols indicate statistical significance (*P* < 0.05).

Abbreviations: BDNF, brain-derived neurotrophic factor; MR, Mendelian randomisation; MR-PRESSO, MR pleiotropy residual sum and outlier; ILAEC, International League Against Epilepsy Consortium.

### 3.3 Causal relationship between plasma BDNF and other neurological disorders

We failed to find strong associations between plasma BDNF levels and other neurological disorders, as shown in Fig. 2 and Table 2. Using the Steiger test, reverse analyses revealed that plasma BDNF levels may be causally unaffected by each specific neurological disease trait (Table S2). The power analysis results are presented in Table S1. There were no indications of directional pleiotropy or heterogeneity, as indicated by *P*_intercept_ > 0.05 and *P*_*Q*_ > 0.05 (Table 4). Scatter, forest, leave-one-out, and funnel plots were generated and are presented in Fig. S1-S22.

### 3.4 Replication and meta-analysis

**Fig. 7:**
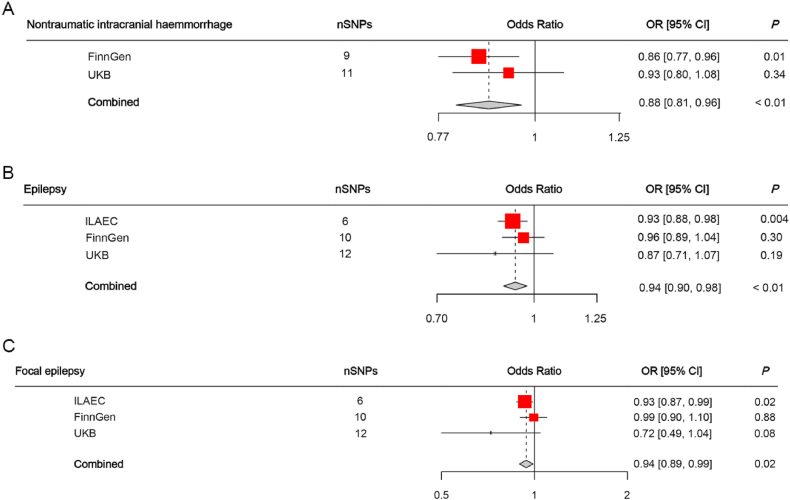
(A) Relations of genetically predicted plasma BDNF with nontraumatic intracranial haemorrhage in the FinnGen consortium and the UKB, as well as combined analyses of both samples. **(B)** Relations of genetically predicted plasma BDNF with epilepsy in the ILAEC, the FinnGen consortium and the UKB, as well as combined analyses about three samples. **(C)** Relations of genetically predicted plasma BDNF with focal epilepsy in the ILAEC, the FinnGen consortium and the UKB, as well as combined analyses of three samples. Abbreviations: SNP, single nucleotide polymorphism; OR, odds ratio; CI, confidence interval; ILAEC, International League Against Epilepsy Consortium; UKB, UK Biobank.

To verify our findings, we conducted replication analyses using GWAS data from the UKB for nITH and from the UKB and FinnGen consortium for epilepsy and focal epilepsy. In line with our expectations, a scatter plot analysis of nITH using UKB GWAS data (Fig. S23) exhibited patterns comparable to those observed in the scatter plot analysis conducted using FinnGen data (Fig. 3(A)). Furthermore, the trends observed in the scatter plots of epilepsy and focal epilepsy in both the FinnGen (Fig. S24 and Fig. S25) and UKB (Fig. S26 and Fig. S27) were consistent with the trends observed in the scatter plots generated by ILAEC (Fig. 3(B) and C). The combined meta-analysis of the FinnGen and UKB datasets further confirmed a significant causal effect of plasma BDNF on nITH (OR = 0.88, 95 % CI: 0.81–0.96, *P* < 0.01) as shown in Fig. 7(A). Additionally, combined analyses of the ILAEC, FinnGen, and UKB datasets revealed a significant causal effect of plasma BDNF on epilepsy (OR = 0.94, 95 % CI: 0.90–0.98, *P* < 0.01) and a suggestive causal impact of plasma BDNF on focal epilepsy (OR = 0.94, 95 % CI: 0.89–0.99, *P* = 0.02), depicted in Fig. 7(B) and (C) respectively.

## 4 Discussion

This study introduced a novel two-sample MR method for evaluating the bidirectional relationship between genetically predicted plasma BDNF levels and various neurological disorders, marking the first instance of such an approach. The findings contribute to our existing understanding by revealing an implied connection of plasma BDNF with nITH. Additionally, our study established a suggestive causal relationship between increased plasma BDNF levels and lower risks of epilepsy and focal epilepsy. However, we failed to find any evidence to support a causal relationship between plasma BDNF and other neurological disorders and related subtypes.

BDNF belongs to the neurotrophin family, which also includes neurotrophins 3 and 4, as well as neural growth factor [3]. *BDNF* is located on human chromosome 11p13 and encodes a full-length protein consisting of 247 amino acids [2]. *BDNF* consists of a common 3′-exon encoding the pro-BDNF region and multiple species-specific 5′-noncoding promoter-adjusted regions, terminating in a coding 5′-exon encompassing gene expression [41]. BDNF is a secreted protein that is initially synthesised in the endoplasmic reticulum as a precursor protein called pre-pro-BDNF and is cleaved into the pro-BDNF isomer (∼32 kDa) when transported to the Golgi apparatus [2]. Pro-BDNF can pass through three different fates: (1) cleaved intracellularly by convertases or furin, resulting in mature BDNF release (∼14 kDa); (2) secreted as pro-BDNF and then cleaved extracellularly by metalloproteinases (MMP) 2, plasmin, and MMP9; and (3) secreted as unmodified pro-BDNF [[42], [43], [44]]. Interestingly, the effects of BDNF are the opposite of those of pro-BDNF in terms of cellular function, adding complexity to the role of BDNF. Mature BDNF binds to TrkB with high affinity, activating phospholipase C-γ (PLCγ) pathway, mitogen-activated protein kinase (MAPK) pathway, phosphoinositide 3-kinase (PI3K) pathway, as well as other biological pathways [45,46]. These pathways result in the phosphorylation and activation of cyclic adenosine monophosphate-response element-binding protein (CREB)-mediated transcription of genes involved in neuronal survival and differentiation [47]. BDNF signalling via activated TrkB receptors is critical for neuronal survival, regeneration, and synaptic formation. It also regulates synaptic structure and function, synaptic plasticity, and maintenance of neural networks [48]. Extracellular pro-BDNF in extracellular fluid may also be bioactive, inducing apoptosis and LTD by binding to the p75 neurotrophin receptor [49]. Pro-BDNF may serve as a signalling factor in the apoptotic cascade. However, whether neurones secrete pro-BDNF under normal conditions remains unclear, as its concentration is relatively lower in presynaptic terminals compared to mature BDNF. The concentration of mature BDNF in animal models can be ten-fold that of pro-BDNF [50], raising doubts about its effectiveness as a signalling molecule. Moreover, BDNF exists in peripheral organs, including the spleen, heart, thymus, and gut [[51], [52], [53]]. Approximately 90 % of BDNF in the blood is maintained within platelets [54]. Circulating BDNF can originate from cerebral and peripheral sources because the blood-brain barrier (BBB) is bidirectionally permeable [55]. Studies in animals and humans have indicated a relationship between the circulating BDNF levels with brain BDNF levels [56].

According to a systematic review and meta-analysis of ischaemic and haemorrhagic stroke, patients with stroke have reduced levels of circulating BDNF compared to those in controls [57]. Pikula et al. (2013) discovered that higher BDNF levels were associated with a reduced risk of stroke and better outcomes [58]. Additionally, BDNF is linked to post-stroke depression (PSD), with lower serum BDNF concentrations associated with an increased risk of PSD [59]. The average serum BDNF level in individuals with haemorrhagic stroke is lower than that in individuals with ischaemic stroke [60]. Based on the exclusion of other brain haemorrhages, nITH can be viewed as equivalent to haemorrhagic stroke [25]. Observational studies support our estimates that an increase of one standard deviation in genetically determined plasma BDNF levels corresponds to a 13.9 % decrease in the risk of nITH, suggesting a protective effect of increased BDNF levels on the disorder. After adjusting for multiple tests, the association did not reach statistical significance (*P*_*FDR*_ = 0.078). However, this suggests that BDNF may be essential for protection against nITH. Serum BDNF levels decrease with stroke severity [60]. This relation becomes more pronounced in individuals with haemorrhagic stroke, as found in a study where the BDNF level of those with intracerebral haemorrhage (ICH) score of 1 was 14.1 ± 3.7 ng/ml, while that of those with ICH score of 3 and above was 5.3 ± 2.3 ng/ml [60]. This evidence, combined with our estimates from the MR analysis, supports the possibility of a causal pathway involving decreased plasma BDNF levels and the progressive pathology of nITH.

Stroke is a severe neurovascular disease characterised by the sudden loss of neurological function owing to a vascular accident [61]. Stroke pathophysiology involves the intricate interaction of multiple molecular mechanisms, such as inflammation, oxidative stress, excitotoxicity, apoptosis, and necrosis [62]. BDNF, through activating downstream pathways like PLCγ, PI3K/Akt, and MAPK/ERK, can protect neurones from apoptosis. This protection mechanism plays a crucial role in preventing the death of neuronal cells and mitigating brain damage during stroke [63,64]. Two pathways prevent apoptosis by interacting with the apoptosis-regulating proteins Bad, BAX, and BCL2. In contrast, all three pathways stimulate the CREB-mediated transcription of pro-survival genes, thereby safeguarding neurones against apoptosis [65]. BDNF may protect neurons from ferroptosis-like cell death by activating nuclear factor-E2-related factor 2 in astrocytes, in addition to its involvement in apoptosis [66]. Furthermore, a novel high-affinity TrkB agonistic antibody has been shown to suppress necroptosis and apoptosis of neuronal cells, reduce infarct size, and facilitate functional recovery in a rat stroke model [67]. Other studies have suggested that BDNF's antinecroptotic effects might be mediated via the PI3K/Akt and MAPK/ERK pathways [68,69]. BDNF reduces the risk of stroke through an additional mechanism, which involves its capacity to enhance neurogenesis. BDNF facilitates the migration of neuronal progenitor cells toward the injury site and promotes their transformation into fully developed neurones [70]. This process is crucial for restoring and regenerating damaged brain tissue and ultimately reducing the risk of stroke. BDNF exhibits anti-inflammatory properties that protect against stroke. In an experimental stroke model, the administration of BDNF through the nasal route was found to increase anti-inflammatory cytokine IL-10 levels but to decrease pro-inflammatory cytokine tumour necrosis factor-alpha levels [71]. In addition, BDNF activates nuclear factor-kappa B, a transcription factor that promotes neuronal survival and inhibiting apoptosis [72]. BDNF mitigates the damage caused by inflammation and reduces the risk of stroke by modulating inflammatory responses. Oxidative pressure, which is related to increased cytosolic calcium levels in response to glutamatergic excitotoxicity, is an early feature of stroke [73]. In an ex vivo brain slice model, BDNF alleviated oxidative stress induced by oxygen and glucose deprivation, by reducing the activity of cytosolic phospholipase A2 [73]. Furthermore, BDNF has been linked to improved sensorimotor recovery and functional outcomes after stroke [70]. It enhances brain plasticity, facilitates the rewiring of neural circuits, and promotes the healing of motor and sensory functions. In humans, the Val66Met (rs6265) SNP affects the intracellular packaging and secretion of BDNF, resulting in learning impairments [74,75]. This SNP affects the clinical course of individuals suffering from ischemic stroke [76] and is related to a poor prognosis in individuals suffering from subarachnoid haemorrhage [77]. The BDNF −196 G > A (Val66Met) polymorphism is linked to early neurological deficits of haemorrhagic stroke, but this effect was not observed in the ischaemic stroke population [78]. Since BDNF plays a vital role in multiple aspects of neurodevelopment, including learning and memory, the BDNF genotype may predict cognitive function following stroke [79]. Therefore, BDNF supplementation may be beneficial in patients with severe stroke symptoms. Several studies have examined the effects of different modalities, including oral or intravenous medications and physical interventions, on BDNF levels. Various drugs such as recombinant tissue plasminogen activator and antioxidants like statins, cytoflavin, and saffron can effectively increase BDNF levels [[80], [81], [82]]. Engaging in physical training provides an immediate boost to BDNF levels; however, its long-term effects do not reach statistical significance [57]. Further research is needed on the optimal dosage of proposed drugs, such as antidepressants and antioxidants, and the appropriate intensity and duration of physical training. In summary, BDNF plays a multifaceted role in reducing the stroke risk by influencing cell death, neurogenesis, inflammation, oxidative stress, and functional recovery. Genetic variations in BDNF function appear to be more closely related to early neurological deficits in patients with haemorrhagic stroke than in those with ischaemic stroke.

Existing reports on changes in BDNF levels in individuals with certain forms of epilepsy, both in the CNS and serum, are inconsistent. Some studies have demonstrated that higher serum BDNF levels are associated with an enhanced course of temporal lobe epilepsy (TLE) and other primary epilepsies [83]. However, a meta-analysis indicated that BDNF levels were not significantly different between patients with epilepsy and control subjects, although patients with partial epilepsy are likely to have lower BDNF levels [17]. Our study showed that a one SD increase in plasma BDNF levels was associated with a 7.3 % reduction in the risk of developing epilepsy. Moreover, higher BDNF levels were associated with reduced odds of non-lesional focal epilepsy and focal epilepsy. The adjusted *P*_*FDR*_ values for epilepsy (0.078), focal epilepsy (*P*_*FDR*_ = 0.139), and non-lesional focal epilepsy (*P*_*FDR*_ = 0.267) did not reach the corrected significance level within any of the evaluated disorders, suggesting that BDNF may be related to epileptic subtypes.

Previous studies have proposed two contrasting hypotheses: BDNF can inhibit or facilitate epileptogenesis [84,85]. One theory suggests that BDNF may promote epileptogenesis by controlling signalling pathways that increase calcium signalling and glutamate expression [84]. Excessive BDNF expression disrupts the regulation of cortical networks in epilepsy. Dysregulation is achieved by enhancing dendritic branching-related plasticity and signalling, resulting in overexcited glutamatergic neurones and lowered seizure thresholds [86]. This results in a positive feedback loop, because elevated production of the neurotransmitter glutamate enhances the expression and signalling of BDNF, thereby further enhancing glutamate signalling. Another hypothesis suggests that BDNF impedes epileptogenesis via a receptor-ligand pathway, particularly involving the TrkB receptor [83]. In an animal study, researchers discovered that BDNF reduced the electrical potential of GABAergic neurones in epileptic rats' brain tissue [87]. BDNF may inhibit epilepsy by phosphorylating specific subunits of GABAergic neurones, thereby attenuating the decrease in GABAergic neuronal excitability associated with epilepsy [87]. BDNF has the potential to mitigate the inflammatory response linked to epileptogenesis, leading to a reduction in BBB disruption and a decrease in spontaneous recurrent seizures in epileptic rat models [88]. Furthermore, phenytoin treatment of epileptic pregnant rats and their offspring increased BDNF levels in the offspring of epileptic rats, demonstrating neuroprotective abilities [89]. The role of BDNF in reducing epilepsy-induced CNS damage is attributed to its neurotrophic properties and subsequent inhibitory effects on epileptogenesis [89]. Furthermore, compelling evidence exists, establishing a strong connection between BDNF-related genes and the development of epilepsy. Patients with TLE have polymorphisms in BDNF-related genes, such as Val66Met, resulting in abnormal BDNF secretion and functional changes [90]. Given the ease and accuracy of testing plasma BDNF levels and rapid acquisition of samples, BDNF has been proposed as a potential peripheral biomarker for evaluating brain health and assessing drug effectiveness. The administration of exogenous BDNF or the enhancement of endogenous BDNF synthesis may offer therapeutic advantages. However, it is crucial to ensure the safe and appropriate use of BDNF by considering factors such as dosage, timing, and spatial context.

We observed null causal relationships between plasma BDNF levels and the majority of disorders investigated, except for nITH, epilepsy, focal epilepsy, and non-lesional focal epilepsy. However, these negative results must be interpreted with caution, because they may be influenced by factors such as limited statistical power and biological complexities. Our study features a relatively large sample size and a two-sample MR method to minimise confounding biases and uses extensive genetic data on neurological disorders. However, our study had several limitations. First, the selection of IVs in this study was based on a *P*-value threshold of 5 × 10^−6^, which could potentially introduce weak instrument bias [91]. Second, although the causal results were promising and innovative, the statistical power for several prevalent neurological disorders was relatively low (Table S1), which diminished the reliability of the findings. This limitation in the ability observed among these disorders was owing to the inadequate sample size, indicating that larger populations are needed to conduct MR studies. Third, genetic variations linked to plasma BDNF levels may be non-optimal when factors such as aging influence the activity of the molecule. BDNF levels tend to decrease with age, potentially owing to the shrinkage of the hippocampus. In older adults, cognitive function may deteriorate as BDNF levels decline [92]. The relationship between decreased BDNF levels and demographic characteristics, particularly age, likely confounds these findings. Antiepileptic medications can decrease BDNF production in both humans and animals [93]. Differences in treatment regimens between patients with partial epilepsy and those with generalized epilepsy may be partly responsible for the lower BDNF levels in patients with partial epilepsy than in controls [17]. These differences in treatment regimens and other potential confounding variables could interfere with the interpretation of causality in the results. To address this issue, further investigation is required to explore the functions of the chosen instrumental variables and develop a more accurate algorithm within the framework of MR. This would assist in eliminating or minimising confounding factors. Fourth, plasma proteins are commonly used as biomarkers in clinical practice owing to their effectiveness and convenience. However, BBB limits the ability to reflect changes in the brain directly and comprehensively. Fifth, as most GWAS data were derived from European populations, caution must be exercised when applying these findings to other ethnic or racial groups. Lastly, while our research indicates a causal relationship between plasma BDNF levels and nITH, epilepsy, and focal epilepsy, it is worth noting that the MR analysis provides forecasting results without empirical verification. Hence, it is imperative to conduct further investigations and validate this causal relationship, along with the underlying pathological mechanism, using animals and humans as test subjects. In future research, we plan to strengthen our research in this area to provide a more comprehensive understanding and clarification of this causal relationship.

## 5 Conclusions

Our MR findings indicate that individuals genetically predisposed to higher plasma levels of BDNF are less likely to develop nITH, epilepsy, or focal epilepsy. Further investigation is warranted to determine how plasma BDNF levels contribute to the pathogenesis of these disorders. This study establishes a theoretical foundation for the development of new and practical therapeutic approaches.

### Ethics declarations

Informed consent was not required for this study because the large-scale GWAS summary statistics of plasma BDNF levels and various neurological disorders used were collected from previous studies. All subjects provided informed consent for all relevant original studies described in the Materials and methods section.

## Funding

This study was funded by grants from the General Program of the 10.13039/501100001809National Natural Science Foundation of China (82274611), the Beijing Municipal Administration of Hospitals' Ascent Plan (DFL20190801), and the 10.13039/501100002799Capital Medical University Xuanwu Hospital's "Talent Cultivation Program" Talent Support Project.

## Data availability statement

Data incorporated in the article, supplementary materials, or cited within the article.

## CRediT authorship contribution statement

**Wei Wang:** Writing – original draft, Methodology, Formal analysis, Conceptualization. **Runshi Gao:** Formal analysis. **Xiaoming Yan:** Writing – review & editing. **Wei Shu:** Writing – review & editing. **Xi Zhang:** Writing – review & editing. **Wenjie Zhang:** Writing – review & editing. **Lan Zhang:** Supervision.

## Declaration of competing interest

The authors declare that they have no known competing financial interests or personal relationships that could have appeared to influence the work reported in this paper.
