# Supplementary material for: Relationship between plasma brain-derived neurotrophic factor levels and neurological disorders: An investigation using Mendelian randomisation
Source: Heliyon. 2024 Apr 26;10(9):e30415. doi: 10.1016/j.heliyon.2024.e30415 (PMC11068855; doi:10.1016/j.heliyon.2024.e30415)
Supplement: Multimedia component 1 [file mmc1.docx]

**Supplementary materials**

**Supplementary tables**

**Table S1** Power calculations for the two-sample MR analysis of plasma BDNF in neurological disorders.

| **Outcome** | **Sample size** | **Proportion of cases** | **OR** | **R^2^** | **Power** |
| --- | --- | --- | --- | --- | --- |
| **Stroke** | 446,696 | 0.09 | 1.027 | 0.069 | 27% |
| Ischemic stroke | 440,328 | 0.08 | 1.020 | 0.069 | 16% |
| Large artery atherosclerosis stroke | 410,484 | 0.01 | 1.080 | 0.069 | 29% |
| Small-vessel stroke | 198,048 | 0.03 | 0.999 | 0.069 | 5% |
| Cardioembolic stroke | 413,304 | 0.02 | 0.992 | 0.069 | 5% |
| Nontraumatic intracranial haemmorrhage | 349,203 | 0.02 | 0.861 | 0.063 | 83% |
| **Neurodegenerative diseases** |  |  |  |  |  |
| Alzheimer’s disease | 377,277 | 0.02 | 1.008 | 0.063 | 5% |
| Early onset Alzheimer’s disease | 171,743 | 0.008 | 0.923 | 0.063 | 11% |
| Late onset Alzheimer’s disease | 176,918 | 0.04 | 1.028 | 0.063 | 9% |
| Parkinson's disease | 482,730 | 0.07 | 1.098 | 0.063 | 99% |
| Amyotrophic lateral sclerosis | 80,610 | 0.26 | 0.982 | 0.078 | 10% |
| Multiple sclerosis | 376,169 | 0.006 | 0.974 | 0.069 | 6% |
| **Epilepsy** |  |  |  |  |  |
| Epilepsy | 44,889 | 0.34 | 0.927 | 0.042 | 34% |
| Focal epilepsy | 39,348 | 0.25 | 0.928 | 0.042 | 25% |
| Focal epilepsy-documented lesion negative | 32,393 | 0.08 | 0.981 | 0.042 | 5% |
| Focal epilepsy-documented hippocampal sclerosis | 30,480 | 0.03 | 0.998 | 0.042 | 5% |
| Focal epilepsy-documented lesion other than hippocampal sclerosis | 32,747 | 0.09 | 0.986 | 0.042 | 5% |
| Generalized epilepsy | 33,446 | 0.11 | 0.932 | 0.042 | 13% |
| Juvenile absence epilepsy | 30,092 | 0.01 | 0.998 | 0.042 | 5% |
| Childhood absence epilepsy | 30,470 | 0.03 | 1.005 | 0.042 | 5% |
| Generalized epilepsy with tonic-clonic seizures | 29,965 | 0.01 | 0.993 | 0.042 | 5% |
| Juvenile myoclonic epilepsy | 30,858 | 0.04 | 0.993 | 0.042 | 5% |
| **Migraine** | 306,314 | 0.06 | 1.008 | 0.069 | 6% |
| Migraine with aura | 295,754 | 0.03 | 1.011 | 0.069 | 6% |
| Migraine without aura, drug-induced | 377,277 | 0.001 | 1.031 | 0.069 | 5% |
| Migraine without aura and triptan purchases | 342,879 | 0.01 | 0.986 | 0.069 | 6% |

The investigation of non-traumatic intracranial haemorrhage utilised the FinnGen consortium data, while analyses of epilepsy, together with its subtypes, relied on data from the ILAEC dataset.

Abbreviations: MR, Mendelian randomisation; BDNF, brain-derived neurotrophic factor; OR, odds ratio; ILAEC, International League Against Epilepsy Consortium.

**Table S2** Steiger direction test of plasma BDNF levels in neurological disorders

| **Outcome** | **Stroke** | **Ischemic stroke** | **Large artery atherosclerosis stroke** | **Small-vessel stroke** | **Cardioembolic stroke** | **Nontraumatic intracranial hemorrhage** | **Alzheimer’s disease** | **Early onset Alzheimer’s disease** | **Late onset Alzheimer’s disease** | **Parkinson’s disease** | **Amyotrophic lateral sclerosis** | **Multiple sclerosis** | **Epilepsy** | **Focal epilepsy** | **Focal epilepsy-documented lesion negative** | **Focal epilepsy-documented hippocampal sclerosis** | **Focal epilepsy-documented lesion other than hippocampal sclerosis** | **Generalized epilepsy** | **Juvenile absence epilepsy** | **Childhood absence epilepsy** | **Generalized epilepsy with tonic-clonic seizures** | **Juvenile myoclonic epilepsy** | **Migraine** | **Migraine with aura** | **Migraine without aura, drug-induced** | **Migraine without aura and triptan purchases** |
| --- | --- | --- | --- | --- | --- | --- | --- | --- | --- | --- | --- | --- | --- | --- | --- | --- | --- | --- | --- | --- | --- | --- | --- | --- | --- | --- |
| Direction | TRUE | TRUE | TRUE | TRUE | TRUE | TRUE | TRUE | TRUE | TRUE | TRUE | TRUE | TRUE | TRUE | TRUE | TRUE | TRUE | TRUE | TRUE | TRUE | TRUE | TRUE | TRUE | TRUE | TRUE | TRUE | TRUE |
| Steiger *P* | 6.42E-50 | 3.32E-50 | 2.52E-49 | 1.13E-47 | 3.87E-49 | 9.31E-44 | 8.00E-45 | 5.90E-44 | 2.51E-44 | 1.43E-44 | 4.80E-50 | 1.45E-49 | 4.40E-25 | 5.76E-25 | 1.03E-24 | 1.57E-24 | 8.50E-26 | 1.25E-23 | 2.49E-25 | 1.87E-25 | 4.54E-23 | 3.47E-24 | 8.52E-50 | 1.48E-48 | 2.16E-49 | 1.66E-49 |

The investigation of non-traumatic intracranial haemorrhage utilised the FinnGen consortium data, while analyses of epilepsy, together with its subtypes, relied on data from the ILAEC dataset. Abbreviations: BDNF, brain-derived neurotrophic factor; ILAEC, International League Against Epilepsy Consortium.

**Table S3** Details of the SNPs removed for possible horizontal pleiotropy

| **SNP** | **Chr** | **Pos.** | **Effect allele** | **Other allele** | **Trait** | **Related disease** | ***P* value** |
| --- | --- | --- | --- | --- | --- | --- | --- |
| rs10015002 | 4 | 109906706 | C | G | Platelet count | Stroke (unspecified) | 2.339 × 10^-13^ |
|  |  |  |  |  |  | Neurodegenerative diseases (unspecified) |  |
|  |  |  |  |  |  | Multiple sclerosis |  |
|  |  |  |  |  |  | Epilepsy (unspecified) |  |
| rs12566888 | 1 | 156899255 | G | T | Platelet count | Stroke (unspecified) | 5.796 × 10^-6^ |
|  |  |  |  |  |  | Neurodegenerative diseases (unspecified) |  |
|  |  |  |  |  |  | Multiple sclerosis |  |
| rs12660365 | 6 | 160388592 | A | G | Coronary artery disease | Stroke (unspecified) | 1.1 × 10^-9^ |
|  |  |  |  |  |  | Neurodegenerative diseases (unspecified) |  |
| rs6993770 | 8 | 105569300 | A | T | Platelet count | Stroke (unspecified) | 1.025 × 10^-67^ |
|  |  |  |  |  |  | Neurodegenerative diseases (unspecified) |  |
|  |  |  |  |  |  | Multiple sclerosis |  |
|  |  |  |  |  |  | Epilepsy (unspecified) |  |

Abbreviations: SNP, single-nucleotide polymorphism; Chr, chromosome; Pos, position; The threshold was set at P < 1×10^-5^.

**Table S4** Harmonised instrumental variables between plasma BDNF levels and neurological disorders

| **Outcome** | **SNP** | **Chr** | **Pos.** | **Effect allele** | **Other allele** | **Exposure** | | | | **Outcome** | | |
| --- | --- | --- | --- | --- | --- | --- | --- | --- | --- | --- | --- | --- |
|  |  |  |  |  |  | **Beta** | **SE** | ***P* value** | **F statistic** | **Beta** | **SE** | ***P* value** |
| Stroke | rs10987903 | 9 | 130926865 | C | G | 0.152 | 0.033 | 4.37E-06 | 24.566 | 0.013 | 0.013 | 0.303 |
|  | rs112183697 | 5 | 160443577 | G | A | -0.245 | 0.050 | 7.59E-07 | 25.007 | -0.022 | 0.021 | 0.292 |
|  | rs117117424 | 12 | 128785456 | C | T | 0.476 | 0.102 | 3.16E-06 | 22.131 | 0.007 | 0.066 | 0.912 |
|  | rs139468884 | 10 | 104581908 | A | G | -0.378 | 0.082 | 3.80E-06 | 21.978 | -0.003 | 0.037 | 0.946 |
|  | rs2063119 | 15 | 98329163 | A | T | 0.120 | 0.026 | 3.09E-06 | 22.580 | 0.000 | 0.010 | 0.967 |
|  | rs4558089 | 10 | 50309330 | C | T | 0.157 | 0.033 | 2.09E-06 | 22.977 | 0.004 | 0.012 | 0.758 |
|  | rs4952755 | 2 | 45447789 | G | C | -0.129 | 0.028 | 3.55E-06 | 22.773 | 0.010 | 0.011 | 0.334 |
|  | rs6676391 | 1 | 240869240 | T | A | 0.281 | 0.060 | 2.69E-06 | 23.487 | 0.003 | 0.024 | 0.902 |
|  | rs72714876 | 15 | 37783271 | T | C | 0.385 | 0.084 | 4.57E-06 | 22.389 | 0.024 | 0.045 | 0.588 |
|  | rs9889896 | 17 | 32343860 | A | C | -0.142 | 0.031 | 3.47E-06 | 22.333 | -0.009 | 0.013 | 0.474 |
| Ischemic stroke | rs10987903 | 9 | 130926865 | C | G | 0.1519 | 0.0331 | 4.37E-06 | 24.5664 | 0.0104 | 0.0137 | 0.448 |
|  | rs112183697 | 5 | 160443577 | G | A | -0.2449 | 0.0495 | 7.59E-07 | 25.0073 | -0.0195 | 0.0223 | 0.3818 |
|  | rs117117424 | 12 | 128785456 | C | T | 0.4755 | 0.1021 | 3.16E-06 | 22.1311 | -0.0075 | 0.073 | 0.9186 |
|  | rs139468884 | 10 | 104581908 | A | G | -0.3777 | 0.0817 | 3.80E-06 | 21.9782 | 0.0157 | 0.0403 | 0.6971 |
|  | rs2063119 | 15 | 98329163 | A | T | 0.1195 | 0.0256 | 3.09E-06 | 22.5797 | 0.004 | 0.0105 | 0.7042 |
|  | rs4558089 | 10 | 50309330 | C | T | 0.1574 | 0.0332 | 2.09E-06 | 22.9768 | 0.0019 | 0.0131 | 0.8862 |
|  | rs4952755 | 2 | 45447789 | G | C | -0.1293 | 0.0279 | 3.55E-06 | 22.7732 | 0.0058 | 0.0116 | 0.6184 |
|  | rs6676391 | 1 | 240869240 | T | A | 0.2808 | 0.0598 | 2.69E-06 | 23.4871 | 0.0014 | 0.0263 | 0.9563 |
|  | rs72714876 | 15 | 37783271 | T | C | 0.3846 | 0.0839 | 4.57E-06 | 22.3888 | 0.009 | 0.045 | 0.8424 |
|  | rs9889896 | 17 | 32343860 | A | C | -0.142 | 0.0306 | 3.47E-06 | 22.3327 | -0.0065 | 0.0139 | 0.6431 |
| Large artery atherosclerosis stroke | rs10987903 | 9 | 130926865 | C | G | 0.1519 | 0.0331 | 4.37E-06 | 24.5664 | -0.0078 | 0.0348 | 0.8222 |
|  | rs112183697 | 5 | 160443577 | G | A | -0.2449 | 0.0495 | 7.59E-07 | 25.0073 | -0.0566 | 0.0596 | 0.3426 |
|  | rs117117424 | 12 | 128785456 | C | T | 0.4755 | 0.1021 | 3.16E-06 | 22.1311 | -0.0233 | 0.2177 | 0.9146 |
|  | rs139468884 | 10 | 104581908 | A | G | -0.3777 | 0.0817 | 3.80E-06 | 21.9782 | 0.1044 | 0.0985 | 0.289 |
|  | rs2063119 | 15 | 98329163 | A | T | 0.1195 | 0.0256 | 3.09E-06 | 22.5797 | 0.0264 | 0.026 | 0.3095 |
|  | rs4558089 | 10 | 50309330 | C | T | 0.1574 | 0.0332 | 2.09E-06 | 22.9768 | 0.0516 | 0.0321 | 0.1081 |
|  | rs4952755 | 2 | 45447789 | G | C | -0.1293 | 0.0279 | 3.55E-06 | 22.7732 | -0.006 | 0.0296 | 0.8391 |
|  | rs6676391 | 1 | 240869240 | T | A | 0.2808 | 0.0598 | 2.69E-06 | 23.4871 | -0.0335 | 0.0692 | 0.6285 |
|  | rs72714876 | 15 | 37783271 | T | C | 0.3846 | 0.0839 | 4.57E-06 | 22.3888 | 0.1708 | 0.1153 | 0.1383 |
|  | rs9889896 | 17 | 32343860 | A | C | -0.142 | 0.0306 | 3.47E-06 | 22.3327 | 0.0169 | 0.0329 | 0.6086 |
| Small-vessel stroke | rs10987903 | 9 | 130926865 | C | G | 0.1519 | 0.0331 | 4.37E-06 | 24.5664 | 0.0324 | 0.0329 | 0.3244 |
|  | rs112183697 | 5 | 160443577 | G | A | -0.2449 | 0.0495 | 7.59E-07 | 25.0073 | 0.093 | 0.0506 | 0.0659 |
|  | rs117117424 | 12 | 128785456 | C | T | 0.4755 | 0.1021 | 3.16E-06 | 22.1311 | 0.1673 | 0.1566 | 0.2855 |
|  | rs139468884 | 10 | 104581908 | A | G | -0.3777 | 0.0817 | 3.80E-06 | 21.9782 | -0.0086 | 0.0994 | 0.9308 |
|  | rs2063119 | 15 | 98329163 | A | T | 0.1195 | 0.0256 | 3.09E-06 | 22.5797 | 0.0078 | 0.0244 | 0.7508 |
|  | rs4558089 | 10 | 50309330 | C | T | 0.1574 | 0.0332 | 2.09E-06 | 22.9768 | 0.0195 | 0.0299 | 0.5147 |
|  | rs4952755 | 2 | 45447789 | G | C | -0.1293 | 0.0279 | 3.55E-06 | 22.7732 | 0.0222 | 0.0272 | 0.4146 |
|  | rs6676391 | 1 | 240869240 | T | A | 0.2808 | 0.0598 | 2.69E-06 | 23.4871 | -0.1269 | 0.0641 | 0.0478 |
|  | rs72714876 | 15 | 37783271 | T | C | 0.3846 | 0.0839 | 4.57E-06 | 22.3888 | 0.0842 | 0.1063 | 0.4284 |
|  | rs9889896 | 17 | 32343860 | A | C | -0.142 | 0.0306 | 3.47E-06 | 22.3327 | -0.0371 | 0.0319 | 0.2443 |
| Cardioembolic stroke | rs10987903 | 9 | 130926865 | C | G | 0.1519 | 0.0331 | 4.37E-06 | 24.5664 | 0.0019 | 0.0268 | 0.9425 |
|  | rs112183697 | 5 | 160443577 | G | A | -0.2449 | 0.0495 | 7.59E-07 | 25.0073 | -0.0887 | 0.0466 | 0.0572 |
|  | rs117117424 | 12 | 128785456 | C | T | 0.4755 | 0.1021 | 3.16E-06 | 22.1311 | 0.1599 | 0.1696 | 0.3459 |
|  | rs139468884 | 10 | 104581908 | A | G | -0.3777 | 0.0817 | 3.80E-06 | 21.9782 | 0.0115 | 0.0827 | 0.8898 |
|  | rs2063119 | 15 | 98329163 | A | T | 0.1195 | 0.0256 | 3.09E-06 | 22.5797 | 6.00E-04 | 0.0199 | 0.9757 |
|  | rs4558089 | 10 | 50309330 | C | T | 0.1574 | 0.0332 | 2.09E-06 | 22.9768 | -0.018 | 0.0255 | 0.4808 |
|  | rs4952755 | 2 | 45447789 | G | C | -0.1293 | 0.0279 | 3.55E-06 | 22.7732 | 0.0355 | 0.0225 | 0.1153 |
|  | rs6676391 | 1 | 240869240 | T | A | 0.2808 | 0.0598 | 2.69E-06 | 23.4871 | 0.0653 | 0.0527 | 0.2149 |
|  | rs72714876 | 15 | 37783271 | T | C | 0.3846 | 0.0839 | 4.57E-06 | 22.3888 | -0.0378 | 0.0878 | 0.6672 |
|  | rs9889896 | 17 | 32343860 | A | C | -0.142 | 0.0306 | 3.47E-06 | 22.3327 | 0.0299 | 0.0265 | 0.2597 |
| Nontraumatic intracranial hemorrhage | rs10987903 | 9 | 130926865 | C | G | 0.152 | 0.033 | 4.37E-06 | 24.566 | -0.001 | 0.023 | 0.966 |
|  | rs112183697 | 5 | 160443577 | G | A | -0.245 | 0.050 | 7.59E-07 | 25.007 | 0.136 | 0.058 | 0.018 |
|  | rs117117424 | 12 | 128785456 | C | T | 0.476 | 0.102 | 3.16E-06 | 22.131 | -0.100 | 0.058 | 0.086 |
|  | rs139468884 | 10 | 104581908 | A | G | -0.378 | 0.082 | 3.80E-06 | 21.978 | -0.026 | 0.152 | 0.867 |
|  | rs2063119 | 15 | 98329163 | A | T | 0.120 | 0.026 | 3.09E-06 | 22.580 | -0.028 | 0.019 | 0.128 |
|  | rs4558089 | 10 | 50309330 | C | T | 0.157 | 0.033 | 2.09E-06 | 22.977 | -0.024 | 0.021 | 0.255 |
|  | rs4952755 | 2 | 45447789 | G | C | -0.129 | 0.028 | 3.55E-06 | 22.773 | 0.011 | 0.019 | 0.571 |
|  | rs6676391 | 1 | 240869240 | T | A | 0.281 | 0.060 | 2.69E-06 | 23.487 | 0.029 | 0.052 | 0.585 |
|  | rs9889896 | 17 | 32343860 | A | C | -0.142 | 0.031 | 3.47E-06 | 22.333 | 0.033 | 0.024 | 0.181 |
| Alzheimer's disease | rs10987903 | 9 | 130926865 | C | G | 0.152 | 0.033 | 4.37E-06 | 24.566 | 0.006 | 0.020 | 0.783 |
|  | rs112183697 | 5 | 160443577 | G | A | -0.245 | 0.050 | 7.59E-07 | 25.007 | 0.034 | 0.051 | 0.499 |
|  | rs117117424 | 12 | 128785456 | C | T | 0.476 | 0.102 | 3.16E-06 | 22.131 | 0.029 | 0.052 | 0.578 |
|  | rs139468884 | 10 | 104581908 | A | G | -0.378 | 0.082 | 3.80E-06 | 21.978 | -0.065 | 0.130 | 0.619 |
|  | rs2063119 | 15 | 98329163 | A | T | 0.120 | 0.026 | 3.09E-06 | 22.580 | -0.022 | 0.017 | 0.179 |
|  | rs4558089 | 10 | 50309330 | C | T | 0.157 | 0.033 | 2.09E-06 | 22.977 | -0.001 | 0.019 | 0.968 |
|  | rs4952755 | 2 | 45447789 | G | C | -0.129 | 0.028 | 3.55E-06 | 22.773 | 0.002 | 0.017 | 0.918 |
|  | rs6676391 | 1 | 240869240 | T | A | 0.281 | 0.060 | 2.69E-06 | 23.487 | 0.005 | 0.046 | 0.916 |
|  | rs9889896 | 17 | 32343860 | A | C | -0.142 | 0.031 | 3.47E-06 | 22.333 | -0.028 | 0.022 | 0.198 |
| Early onset Alzheimer’s disease | rs10987903 | 9 | 130926865 | C | G | 0.152 | 0.033 | 4.37E-06 | 24.566 | 0.050 | 0.051 | 0.322 |
|  | rs112183697 | 5 | 160443577 | G | A | -0.245 | 0.050 | 7.59E-07 | 25.007 | 0.111 | 0.129 | 0.390 |
|  | rs117117424 | 12 | 128785456 | C | T | 0.476 | 0.102 | 3.16E-06 | 22.131 | -0.068 | 0.132 | 0.605 |
|  | rs139468884 | 10 | 104581908 | A | G | -0.378 | 0.082 | 3.80E-06 | 21.978 | -0.032 | 0.344 | 0.926 |
|  | rs2063119 | 15 | 98329163 | A | T | 0.120 | 0.026 | 3.09E-06 | 22.580 | 0.011 | 0.042 | 0.800 |
|  | rs4558089 | 10 | 50309330 | C | T | 0.157 | 0.033 | 2.09E-06 | 22.977 | -0.031 | 0.048 | 0.516 |
|  | rs4952755 | 2 | 45447789 | G | C | -0.129 | 0.028 | 3.55E-06 | 22.773 | 0.003 | 0.043 | 0.948 |
|  | rs6676391 | 1 | 240869240 | T | A | 0.281 | 0.060 | 2.69E-06 | 23.487 | -0.019 | 0.117 | 0.870 |
|  | rs9889896 | 17 | 32343860 | A | C | -0.142 | 0.031 | 3.47E-06 | 22.333 | 0.063 | 0.055 | 0.254 |
| Late onset Alzheimer’s disease | rs10987903 | 9 | 130926865 | C | G | 0.152 | 0.033 | 4.37E-06 | 24.566 | -0.007 | 0.026 | 0.780 |
|  | rs112183697 | 5 | 160443577 | G | A | -0.245 | 0.050 | 7.59E-07 | 25.007 | 0.034 | 0.065 | 0.602 |
|  | rs117117424 | 12 | 128785456 | C | T | 0.476 | 0.102 | 3.16E-06 | 22.131 | 0.042 | 0.067 | 0.531 |
|  | rs139468884 | 10 | 104581908 | A | G | -0.378 | 0.082 | 3.80E-06 | 21.978 | -0.119 | 0.171 | 0.488 |
|  | rs2063119 | 15 | 98329163 | A | T | 0.120 | 0.026 | 3.09E-06 | 22.580 | 0.002 | 0.021 | 0.944 |
|  | rs4558089 | 10 | 50309330 | C | T | 0.157 | 0.033 | 2.09E-06 | 22.977 | -0.010 | 0.024 | 0.668 |
|  | rs4952755 | 2 | 45447789 | G | C | -0.129 | 0.028 | 3.55E-06 | 22.773 | -0.003 | 0.022 | 0.893 |
|  | rs6676391 | 1 | 240869240 | T | A | 0.281 | 0.060 | 2.69E-06 | 23.487 | 0.021 | 0.059 | 0.716 |
|  | rs9889896 | 17 | 32343860 | A | C | -0.142 | 0.031 | 3.47E-06 | 22.333 | -0.026 | 0.028 | 0.351 |
| Parkinson's disease | rs10987903 | 9 | 130926865 | C | G | 0.1519 | 0.0331 | 4.37E-06 | 24.5664 | 0.007 | 0.0293 | 0.8102 |
|  | rs112183697 | 5 | 160443577 | G | A | -0.2449 | 0.0495 | 7.59E-07 | 25.0073 | -0.0453 | 0.0472 | 0.3377 |
|  | rs117117424 | 12 | 128785456 | C | T | 0.4755 | 0.1021 | 3.16E-06 | 22.1311 | 0.0966 | 0.0997 | 0.3326 |
|  | rs139468884 | 10 | 104581908 | A | G | -0.3777 | 0.0817 | 3.80E-06 | 21.9782 | -0.0172 | 0.062 | 0.7813 |
|  | rs2063119 | 15 | 98329163 | A | T | 0.1195 | 0.0256 | 3.09E-06 | 22.5797 | -0.0225 | 0.0229 | 0.326 |
|  | rs4558089 | 10 | 50309330 | C | T | 0.1574 | 0.0332 | 2.09E-06 | 22.9768 | 0.01 | 0.0223 | 0.6533 |
|  | rs4952755 | 2 | 45447789 | G | C | -0.1293 | 0.0279 | 3.55E-06 | 22.7732 | -0.0125 | 0.0254 | 0.6236 |
|  | rs6676391 | 1 | 240869240 | T | A | 0.2808 | 0.0598 | 2.69E-06 | 23.4871 | 0.0889 | 0.056 | 0.1126 |
|  | rs72714876 | 15 | 37783271 | T | C | 0.3846 | 0.0839 | 4.57E-06 | 22.3888 | 0.0664 | 0.086 | 0.4401 |
| Amyotrophic lateral sclerosis | rs10987903 | 9 | 130926865 | C | G | 0.1519 | 0.0331 | 4.37E-06 | 24.5664 | -0.0088 | 0.0183 | 0.6292 |
|  | rs112183697 | 5 | 160443577 | G | A | -0.2449 | 0.0495 | 7.59E-07 | 25.0073 | 0.0158 | 0.0294 | 0.592 |
|  | rs117117424 | 12 | 128785456 | C | T | 0.4755 | 0.1021 | 3.16E-06 | 22.1311 | -0.0799 | 0.1358 | 0.5561 |
|  | rs139468884 | 10 | 104581908 | A | G | -0.3777 | 0.0817 | 3.80E-06 | 21.9782 | 0.0131 | 0.0498 | 0.793 |
|  | rs2063119 | 15 | 98329163 | A | T | 0.1195 | 0.0256 | 3.09E-06 | 22.5797 | -0.0047 | 0.0141 | 0.7413 |
|  | rs4558089 | 10 | 50309330 | C | T | 0.1574 | 0.0332 | 2.09E-06 | 22.9768 | -0.024 | 0.0177 | 0.1742 |
|  | rs4952755 | 2 | 45447789 | G | C | -0.1293 | 0.0279 | 3.55E-06 | 22.7732 | 0.0075 | 0.0159 | 0.6387 |
|  | rs558293908 | 14 | 68995402 | G | T | -0.1548 | 0.0321 | 1.45E-06 | 27.8228 | -0.0354 | 0.0296 | 0.2314 |
|  | rs6676391 | 1 | 240869240 | T | A | 0.2808 | 0.0598 | 2.69E-06 | 23.4871 | -0.0124 | 0.0329 | 0.7070 |
|  | rs72714876 | 15 | 37783271 | T | C | 0.3846 | 0.0839 | 4.57E-06 | 22.3888 | 0.165 | 0.0578 | 0.0043 |
|  | rs9889896 | 17 | 32343860 | A | C | -0.142 | 0.0306 | 3.47E-06 | 22.3327 | 0.0059 | 0.0206 | 0.7748 |
| Multiple sclerosis | rs10987903 | 9 | 130926865 | C | G | 0.152 | 0.0331 | 4.37E-06 | 24.5664 | 0.005 | 0.039 | 0.897 |
|  | rs112183697 | 5 | 160443577 | G | A | -0.245 | 0.0495 | 7.59E-07 | 25.0073 | -0.132 | 0.098 | 0.178 |
|  | rs117117424 | 12 | 128785456 | C | T | 0.476 | 0.1021 | 3.16E-06 | 22.1311 | -0.089 | 0.101 | 0.378 |
|  | rs12660365 | 6 | 160809624 | A | G | 0.157 | 0.0338 | 3.39E-06 | 22.0405 | 0.007 | 0.038 | 0.851 |
|  | rs139468884 | 10 | 104581908 | A | G | -0.378 | 0.0817 | 3.80E-06 | 21.9782 | -0.155 | 0.247 | 0.531 |
|  | rs2063119 | 15 | 98329163 | A | T | 0.120 | 0.0256 | 3.09E-06 | 22.5797 | -0.028 | 0.032 | 0.377 |
|  | rs4558089 | 10 | 50309330 | C | T | 0.157 | 0.0332 | 2.09E-06 | 22.9768 | 0.000 | 0.037 | 0.990 |
|  | rs4952755 | 2 | 45447789 | G | C | -0.129 | 0.0279 | 3.55E-06 | 22.7732 | -0.034 | 0.033 | 0.305 |
|  | rs6676391 | 1 | 240869240 | T | A | 0.281 | 0.0598 | 2.69E-06 | 23.4871 | -0.085 | 0.086 | 0.324 |
|  | rs9889896 | 17 | 32343860 | A | C | -0.142 | 0.0306 | 3.47E-06 | 22.3327 | 0.031 | 0.042 | 0.456 |
| Epilepsy | rs112183697 | 5 | 160443577 | G | A | -0.2449 | 0.0495 | 7.59E-07 | 25.0073 | 0.0265 | 0.0160 | 0.0980 |
|  | rs12660365 | 6 | 160809624 | A | G | 0.1569 | 0.0338 | 3.39E-06 | 22.0405 | -0.0177 | 0.0102 | 0.0810 |
|  | rs139468884 | 10 | 104581908 | A | G | -0.3777 | 0.0817 | 3.80E-06 | 21.9782 | 0.0114 | 0.0286 | 0.6900 |
|  | rs2063119 | 15 | 98329163 | A | T | 0.1195 | 0.0256 | 3.09E-06 | 22.5797 | -0.0115 | 0.0078 | 0.1400 |
|  | rs4558089 | 10 | 50309330 | C | T | 0.1574 | 0.0332 | 2.09E-06 | 22.9768 | 0.0027 | 0.0093 | 0.7743 |
|  | rs6676391 | 1 | 240869240 | T | A | 0.2808 | 0.0598 | 2.69E-06 | 23.4871 | -0.0361 | 0.0172 | 0.0358 |
| Focal epilepsy | rs112183697 | 5 | 160443577 | G | A | -0.2449 | 0.0495 | 7.59E-07 | 25.0073 | 0.0180 | 0.0189 | 0.3400 |
|  | rs12660365 | 6 | 160809624 | A | G | 0.1569 | 0.0338 | 3.39E-06 | 22.0405 | -0.0225 | 0.0119 | 0.0580 |
|  | rs139468884 | 10 | 104581908 | A | G | -0.3777 | 0.0817 | 3.80E-06 | 21.9782 | 0.0553 | 0.0331 | 0.0950 |
|  | rs2063119 | 15 | 98329163 | A | T | 0.1195 | 0.0256 | 3.09E-06 | 22.5797 | -0.0112 | 0.0091 | 0.2200 |
|  | rs4558089 | 10 | 50309330 | C | T | 0.1574 | 0.0332 | 2.09E-06 | 22.9768 | 0.0073 | 0.0108 | 0.4998 |
|  | rs6676391 | 1 | 240869240 | T | A | 0.2808 | 0.0598 | 2.69E-06 | 23.4871 | -0.0219 | 0.0197 | 0.2655 |
| Focal epilepsy-documented lesion negative | rs112183697 | 5 | 160443577 | G | A | -0.2449 | 0.0495 | 7.59E-07 | 25.0073 | 0.0026 | 0.0056 | 0.6400 |
|  | rs12660365 | 6 | 160809624 | A | G | 0.1569 | 0.0338 | 3.39E-06 | 22.0405 | -0.0056 | 0.0035 | 0.1100 |
|  | rs139468884 | 10 | 104581908 | A | G | -0.3777 | 0.0817 | 3.80E-06 | 21.9782 | 0.0178 | 0.0097 | 0.0660 |
|  | rs2063119 | 15 | 98329163 | A | T | 0.1195 | 0.0256 | 3.09E-06 | 22.5797 | -0.0037 | 0.0027 | 0.1600 |
|  | rs4558089 | 10 | 50309330 | C | T | 0.1574 | 0.0332 | 2.09E-06 | 22.9768 | 0.0001 | 0.0033 | 0.9800 |
|  | rs6676391 | 1 | 240869240 | T | A | 0.2808 | 0.0598 | 2.69E-06 | 23.4871 | 0.0005 | 0.0061 | 0.9400 |
| Focal epilepsy-documented hippocampal sclerosis | rs112183697 | 5 | 160443577 | G | A | -0.2449 | 0.0495 | 7.59E-07 | 25.0073 | 0.0027 | 0.0032 | 0.3900 |
|  | rs12660365 | 6 | 160809624 | A | G | 0.1569 | 0.0338 | 3.39E-06 | 22.0405 | -0.0042 | 0.0020 | 0.0330 |
|  | rs139468884 | 10 | 104581908 | A | G | -0.3777 | 0.0817 | 3.80E-06 | 21.9782 | 0.0004 | 0.0055 | 0.9500 |
|  | rs2063119 | 15 | 98329163 | A | T | 0.1195 | 0.0256 | 3.09E-06 | 22.5797 | 0.0022 | 0.0015 | 0.1500 |
|  | rs4558089 | 10 | 50309330 | C | T | 0.1574 | 0.0332 | 2.09E-06 | 22.9768 | 0.0012 | 0.0019 | 0.5100 |
|  | rs6676391 | 1 | 240869240 | T | A | 0.2808 | 0.0598 | 2.69E-06 | 23.4871 | 0.0004 | 0.0035 | 0.9000 |
| Focal epilepsy-documented lesion other than hippocampal sclerosis | rs112183697 | 5 | 160443577 | G | A | -0.2449 | 0.0495 | 7.59E-07 | 25.0073 | -0.0007 | 0.0056 | 0.89 |
|  | rs12660365 | 6 | 160809624 | A | G | 0.1569 | 0.0338 | 3.39E-06 | 22.0405 | -0.0046 | 0.0035 | 0.19 |
|  | rs139468884 | 10 | 104581908 | A | G | -0.3777 | 0.0817 | 3.80E-06 | 21.9782 | 0.0069 | 0.0099 | 0.48 |
|  | rs2063119 | 15 | 98329163 | A | T | 0.1195 | 0.0256 | 3.09E-06 | 22.5797 | -0.0029 | 0.0027 | 0.28 |
|  | rs4558089 | 10 | 50309330 | C | T | 0.1574 | 0.0332 | 2.09E-06 | 22.9768 | 0.0005 | 0.0034 | 0.89 |
|  | rs6676391 | 1 | 240869240 | T | A | 0.2808 | 0.0598 | 2.69E-06 | 23.4871 | -0.0062 | 0.0062 | 0.32 |
| Generalized epilepsy | rs112183697 | 5 | 160443577 | G | A | -0.2449 | 0.0495 | 7.59E-07 | 25.0073 | 0.0491 | 0.0271 | 0.0700 |
|  | rs12660365 | 6 | 160809624 | A | G | 0.1569 | 0.0338 | 3.39E-06 | 22.0405 | -0.0004 | 0.0170 | 0.9800 |
|  | rs139468884 | 10 | 104581908 | A | G | -0.3777 | 0.0817 | 3.80E-06 | 21.9782 | -0.0890 | 0.0482 | 0.0650 |
|  | rs2063119 | 15 | 98329163 | A | T | 0.1195 | 0.0256 | 3.09E-06 | 22.5797 | -0.0183 | 0.0131 | 0.1600 |
|  | rs4558089 | 10 | 50309330 | C | T | 0.1574 | 0.0332 | 2.09E-06 | 22.9768 | 0.0028 | 0.0160 | 0.8633 |
|  | rs6676391 | 1 | 240869240 | T | A | 0.2808 | 0.0598 | 2.69E-06 | 23.4871 | -0.0704 | 0.0303 | 0.0200 |
| Juvenile absence epilepsy | rs112183697 | 5 | 160443577 | G | A | -0.2449 | 0.0495 | 7.59E-07 | 25.0073 | -0.0001 | 0.0025 | 0.97 |
|  | rs12660365 | 6 | 160809624 | A | G | 0.1569 | 0.0338 | 3.39E-06 | 22.0405 | 0.0011 | 0.0016 | 0.47 |
|  | rs139468884 | 10 | 104581908 | A | G | -0.3777 | 0.0817 | 3.80E-06 | 21.9782 | -0.0046 | 0.0044 | 0.29 |
|  | rs2063119 | 15 | 98329163 | A | T | 0.1195 | 0.0256 | 3.09E-06 | 22.5797 | -0.0009 | 0.0012 | 0.43 |
|  | rs4558089 | 10 | 50309330 | C | T | 0.1574 | 0.0332 | 2.09E-06 | 22.9768 | -0.0008 | 0.0015 | 0.58 |
|  | rs6676391 | 1 | 240869240 | T | A | 0.2808 | 0.0598 | 2.69E-06 | 23.4871 | -0.0042 | 0.0028 | 0.13 |
| Childhood absence epilepsy | rs112183697 | 5 | 160443577 | G | A | -0.2449 | 0.0495 | 7.59E-07 | 25.0073 | 0.0020 | 0.0034 | 0.5400 |
|  | rs12660365 | 6 | 160809624 | A | G | 0.1569 | 0.0338 | 3.39E-06 | 22.0405 | 0.0001 | 0.0021 | 0.9700 |
|  | rs139468884 | 10 | 104581908 | A | G | -0.3777 | 0.0817 | 3.80E-06 | 21.9782 | -0.0056 | 0.0059 | 0.3400 |
|  | rs2063119 | 15 | 98329163 | A | T | 0.1195 | 0.0256 | 3.09E-06 | 22.5797 | -0.0003 | 0.0016 | 0.8400 |
|  | rs4558089 | 10 | 50309330 | C | T | 0.1574 | 0.0332 | 2.09E-06 | 22.9768 | 0.0036 | 0.0020 | 0.0700 |
|  | rs6676391 | 1 | 240869240 | T | A | 0.2808 | 0.0598 | 2.69E-06 | 23.4871 | 0.0000 | 0.0037 | 0.9900 |
| Generalized epilepsy with tonic-clonic seizures | rs112183697 | 5 | 160443577 | G | A | -0.2449 | 0.0495 | 7.59E-07 | 25.0073 | 0.0042 | 0.0019 | 0.0240 |
|  | rs12660365 | 6 | 160809624 | A | G | 0.1569 | 0.0338 | 3.39E-06 | 22.0405 | -0.0008 | 0.0012 | 0.5200 |
|  | rs139468884 | 10 | 104581908 | A | G | -0.3777 | 0.0817 | 3.80E-06 | 21.9782 | 0.0055 | 0.0033 | 0.0950 |
|  | rs2063119 | 15 | 98329163 | A | T | 0.1195 | 0.0256 | 3.09E-06 | 22.5797 | -0.0015 | 0.0009 | 0.0890 |
|  | rs4558089 | 10 | 50309330 | C | T | 0.1574 | 0.0332 | 2.09E-06 | 22.9768 | 0.0018 | 0.0011 | 0.1200 |
|  | rs6676391 | 1 | 240869240 | T | A | 0.2808 | 0.0598 | 2.69E-06 | 23.4871 | -0.0018 | 0.0021 | 0.3800 |
| Juvenile myoclonic epilepsy | rs112183697 | 5 | 160443577 | G | A | -0.2449 | 0.0495 | 7.59E-07 | 25.0073 | 0.0026 | 0.0040 | 0.5200 |
|  | rs12660365 | 6 | 160809624 | A | G | 0.1569 | 0.0338 | 3.39E-06 | 22.0405 | 0.0003 | 0.0025 | 0.9000 |
|  | rs139468884 | 10 | 104581908 | A | G | -0.3777 | 0.0817 | 3.80E-06 | 21.9782 | -0.0125 | 0.0071 | 0.0780 |
|  | rs2063119 | 15 | 98329163 | A | T | 0.1195 | 0.0256 | 3.09E-06 | 22.5797 | -0.0022 | 0.0019 | 0.2500 |
|  | rs4558089 | 10 | 50309330 | C | T | 0.1574 | 0.0332 | 2.09E-06 | 22.9768 | -0.0008 | 0.0024 | 0.7500 |
|  | rs6676391 | 1 | 240869240 | T | A | 0.2808 | 0.0598 | 2.69E-06 | 23.4871 | -0.0095 | 0.0044 | 0.0330 |
| Migraine | rs10987903 | 9 | 130926865 | C | G | 0.152 | 0.033 | 4.37E-06 | 24.566 | 0.0146 | 0.014 | 0.297 |
|  | rs112183697 | 5 | 160443577 | G | A | -0.245 | 0.050 | 7.59E-07 | 25.007 | 0.0038 | 0.035 | 0.914 |
|  | rs117117424 | 12 | 128785456 | C | T | 0.476 | 0.102 | 3.16E-06 | 22.131 | -0.0020 | 0.036 | 0.956 |
|  | rs12660365 | 6 | 160809624 | A | G | 0.157 | 0.034 | 3.39E-06 | 22.040 | 0.0068 | 0.013 | 0.612 |
|  | rs139468884 | 10 | 104581908 | A | G | -0.378 | 0.082 | 3.80E-06 | 21.978 | -0.0002 | 0.094 | 0.998 |
|  | rs2063119 | 15 | 98329163 | A | T | 0.120 | 0.026 | 3.09E-06 | 22.580 | -0.0005 | 0.011 | 0.969 |
|  | rs4558089 | 10 | 50309330 | C | T | 0.157 | 0.033 | 2.09E-06 | 22.977 | 0.0072 | 0.013 | 0.582 |
|  | rs4952755 | 2 | 45447789 | G | C | -0.129 | 0.028 | 3.55E-06 | 22.773 | 0.0120 | 0.012 | 0.312 |
|  | rs6676391 | 1 | 240869240 | T | A | 0.281 | 0.060 | 2.69E-06 | 23.487 | 0.0118 | 0.032 | 0.714 |
|  | rs9889896 | 17 | 32343860 | A | C | -0.142 | 0.031 | 3.47E-06 | 22.333 | 0.0094 | 0.015 | 0.531 |
| Migraine with aura | rs10987903 | 9 | 130926865 | C | G | 0.152 | 0.033 | 4.37E-06 | 24.566 | 0.033 | 0.021 | 0.110 |
|  | rs112183697 | 5 | 160443577 | G | A | -0.245 | 0.050 | 7.59E-07 | 25.007 | 0.057 | 0.053 | 0.281 |
|  | rs117117424 | 12 | 128785456 | C | T | 0.476 | 0.102 | 3.16E-06 | 22.131 | -0.063 | 0.054 | 0.238 |
|  | rs12660365 | 6 | 160809624 | A | G | 0.157 | 0.034 | 3.39E-06 | 22.040 | 0.034 | 0.020 | 0.090 |
|  | rs139468884 | 10 | 104581908 | A | G | -0.378 | 0.082 | 3.80E-06 | 21.978 | -0.013 | 0.142 | 0.926 |
|  | rs2063119 | 15 | 98329163 | A | T | 0.120 | 0.026 | 3.09E-06 | 22.580 | -0.001 | 0.017 | 0.952 |
|  | rs4558089 | 10 | 50309330 | C | T | 0.157 | 0.033 | 2.09E-06 | 22.977 | 0.014 | 0.019 | 0.480 |
|  | rs4952755 | 2 | 45447789 | G | C | -0.129 | 0.028 | 3.55E-06 | 22.773 | 0.022 | 0.018 | 0.201 |
|  | rs6676391 | 1 | 240869240 | T | A | 0.281 | 0.060 | 2.69E-06 | 23.487 | 0.055 | 0.048 | 0.259 |
|  | rs9889896 | 17 | 32343860 | A | C | -0.142 | 0.031 | 3.47E-06 | 22.333 | 0.026 | 0.022 | 0.248 |
| Migraine without aura, drug-induced | rs10987903 | 9 | 130926865 | C | G | 0.152 | 0.033 | 4.37E-06 | 24.566 | -0.135 | 0.101 | 0.181 |
|  | rs112183697 | 5 | 160443577 | G | A | -0.245 | 0.050 | 7.59E-07 | 25.007 | -0.071 | 0.257 | 0.783 |
|  | rs117117424 | 12 | 128785456 | C | T | 0.476 | 0.102 | 3.16E-06 | 22.131 | -0.297 | 0.257 | 0.249 |
|  | rs12660365 | 6 | 160809624 | A | G | 0.157 | 0.034 | 3.39E-06 | 22.040 | 0.029 | 0.095 | 0.763 |
|  | rs139468884 | 10 | 104581908 | A | G | -0.378 | 0.082 | 3.80E-06 | 21.978 | -0.474 | 0.739 | 0.521 |
|  | rs2063119 | 15 | 98329163 | A | T | 0.120 | 0.026 | 3.09E-06 | 22.580 | 0.083 | 0.082 | 0.316 |
|  | rs4558089 | 10 | 50309330 | C | T | 0.157 | 0.033 | 2.09E-06 | 22.977 | 0.038 | 0.094 | 0.685 |
|  | rs4952755 | 2 | 45447789 | G | C | -0.129 | 0.028 | 3.55E-06 | 22.773 | -0.043 | 0.086 | 0.613 |
|  | rs6676391 | 1 | 240869240 | T | A | 0.281 | 0.060 | 2.69E-06 | 23.487 | -0.229 | 0.244 | 0.348 |
|  | rs9889896 | 17 | 32343860 | A | C | -0.142 | 0.031 | 3.47E-06 | 22.333 | -0.150 | 0.108 | 0.163 |
| Migraine without aura and triptan purchases | rs10987903 | 9 | 130926865 | C | G | 0.152 | 0.033 | 4.37E-06 | 24.566 | -0.016 | 0.026 | 0.530 |
|  | rs112183697 | 5 | 160443577 | G | A | -0.245 | 0.050 | 7.59E-07 | 25.007 | 0.027 | 0.066 | 0.680 |
|  | rs117117424 | 12 | 128785456 | C | T | 0.476 | 0.102 | 3.16E-06 | 22.131 | 0.042 | 0.067 | 0.538 |
|  | rs12660365 | 6 | 160809624 | A | G | 0.157 | 0.034 | 3.39E-06 | 22.040 | -0.029 | 0.025 | 0.249 |
|  | rs139468884 | 10 | 104581908 | A | G | -0.378 | 0.082 | 3.80E-06 | 21.978 | 0.039 | 0.176 | 0.823 |
|  | rs2063119 | 15 | 98329163 | A | T | 0.120 | 0.026 | 3.09E-06 | 22.580 | 0.031 | 0.021 | 0.153 |
|  | rs4558089 | 10 | 50309330 | C | T | 0.157 | 0.033 | 2.09E-06 | 22.977 | -0.020 | 0.025 | 0.419 |
|  | rs4952755 | 2 | 45447789 | G | C | -0.129 | 0.028 | 3.55E-06 | 22.773 | -0.019 | 0.022 | 0.402 |
|  | rs6676391 | 1 | 240869240 | T | A | 0.281 | 0.060 | 2.69E-06 | 23.487 | -0.012 | 0.061 | 0.849 |
|  | rs9889896 | 17 | 32343860 | A | C | -0.142 | 0.031 | 3.47E-06 | 22.333 | 0.013 | 0.028 | 0.654 |

The investigation of non-traumatic intracranial haemorrhage utilised the FinnGen consortium data, while analyses of epilepsy as well as its subtypes relied on data from the ILAEC dataset. Abbreviations: BDNF, brain-derived neurotrophic factor; SNP, single-nucleotide polymorphism; Chr, chromosome; Pos, position; SE, standard error; ILAEC, International League Against Epilepsy Consortium.

**Table S5** Detailed information on the studies used

| **Phenotype** | **Consortium** | **Sample size**  **(cases/controls）** | **Population** | **Journal** | **Year** | **References** |
| --- | --- | --- | --- | --- | --- | --- |
| Intracranial hemorrhage | UKB | 2,437/407,633 | European | - | 2018 | - |
| Epilepsy | FinnGen | 11,740/287,837 | European | - | 2023 | - |
|  | UKB | 1,128/403,249 | European | - | 2018 |  |
| Focal epilepsy | FinnGen | 6,213/365,534 | European | - | 2023 | - |
|  | UKB | 345/403,249 | European | - | 2018 | - |

Abbreviations: UKB, UK Biobank

**Table S6** Relationship between plasma BDNF and intracranial haemorrhage, epilepsy, and focal epilepsy

| **Outcome** | | **IVW-random effects** | | | **MR-Egger** | | | **Weighted median** | | | **Weighted mode** | | | **Cochrane’s Q test** | | **MR-Egger Intercept test** | | **MR-PRESSO global test** |
| --- | --- | --- | --- | --- | --- | --- | --- | --- | --- | --- | --- | --- | --- | --- | --- | --- | --- | --- |
|  |  | **Beta** | **OR (95%CI)** | ***P* value** | **Beta** | **OR (95%CI)** | ***P* value** | **Beta** | **OR (95%CI)** | ***P* value** | **Beta** | **OR (95%CI)** | ***P* value** | **Q-value** | ***P_Q_*** | **Intercept** | ***P*_intercept_** | ***P* value** |
| Intracranial hemorrhage | UKB | -0.072 | 0.931 (0.803, 1.078) | 0.337 | -0.034 | 0.966 (0.652, 1.432) | 0.868 | -0.096 | 0.908 (0.746, 1.106) | 0.34 | -0.151 | 0.86 (0.63, 1.174) | 0.364 | 3.735 | 0.959 | -0.007 | 0.844 | 0.959 |
| Epilepsy | FinnGen | -0.039 | 0.962 (0.893, 1.036) | 0.302 | -0.169 | 0.844 (0.679, 1.049) | 0.165 | -0.031 | 0.970 (0.880, 1.068) | 0.535 | -0.033 | 0.967 (0.841, 1.112) | 0.650 | 5.285 | 0.809 | 0.023 | 0.246 | 0.806 |
|  | UKB | -0.138 | 0.871 (0.709, 1.071) | 0.191 | -0.075 | 0.928 (0.522, 1.650) | 0.805 | -0.134 | 0.875 (0.671, 1.140) | 0.322 | -0.105 | 0.900 (0.591, 1.370) | 0.633 | 6.553 | 0.834 | -0.012 | 0.822 | 0.832 |
| Focal epilepsy | FinnGen | -0.008 | 0.993 (0.897, 1.098) | 0.884 | -0.131 | 0.877 (0.654, 1.176) | 0.406 | 0.028 | 1.029 (0.908, 1.166) | 0.657 | 0.027 | 1.027 (0.866, 1.220) | 0.764 | 8.189 | 0.515 | 0.022 | 0.404 | 0.575 |
|  | UKB | -0.329 | 0.719 (0.495, 1.045) | 0.084 | -0.369 | 0.691 (0.244, 1.956） | 0.503 | -0.396 | 0.673 (0.409, 1.106) | 0.118 | -0.476 | 0.622 (0.274, 1.409) | 0.279 | 6.232 | 0.857 | 0.007 | 0.937 | 0.865 |

Abbreviations: BDNF, brain-derived neurotrophic factor; IVW, inverse-variance weighted; MR, Mendelian randomisation; OR, odds ratio; CI, confidence interval; UKB, UK Biobank.

**Table S7** Harmonised instrumental variables between plasma BDNF levels and intracranial haemorrhage, epilepsy, and focal epilepsy.

| **Outcome** | **SNP** | **Chr** | **Pos.** | **Effect allele** | **Other allele** | **Exposure** | | | | **Outcome** | | |
| --- | --- | --- | --- | --- | --- | --- | --- | --- | --- | --- | --- | --- |
|  |  |  |  |  |  | **Beta** | **SE** | ***P* value** | **F statistic** | **Beta** | **SE** | ***P* value** |
| Intracranial hemorrhage (UKB) | rs10987903 | 9 | 130926865 | C | G | 0.152 | 0.033 | 4.37E-06 | 24.566 | -0.041 | 0.037 | 0.563 |
|  | rs112183697 | 5 | 160443577 | G | A | -0.245 | 0.050 | 7.59E-07 | 25.007 | 0.051 | 0.058 | 0.423 |
|  | rs117117424 | 12 | 128785456 | C | T | 0.476 | 0.102 | 3.16E-06 | 22.131 | -0.097 | 0.118 | 0.385 |
|  | rs139468884 | 10 | 104581908 | A | G | -0.378 | 0.082 | 3.80E-06 | 21.978 | 0.026 | 0.107 | 0.092 |
|  | rs2063119 | 15 | 98329163 | A | T | 0.120 | 0.026 | 3.09E-06 | 22.580 | -0.024 | 0.030 | 0.368 |
|  | rs4558089 | 10 | 50309330 | C | T | 0.157 | 0.033 | 2.09E-06 | 22.977 | -0.015 | 0.039 | 0.156 |
|  | rs4952755 | 2 | 45447789 | G | C | -0.129 | 0.028 | 3.55E-06 | 22.773 | -0.001 | 0.032 | 0.014 |
|  | rs558293908 | 14 | 68995402 | G | T | -0.155 | 0.032 | 1.45E-06 | 27.823 | -0.021 | 0.037 | 0.249 |
|  | rs6676391 | 1 | 240869240 | T | A | 0.281 | 0.060 | 2.69E-06 | 23.487 | 0.055 | 0.068 | 0.377 |
|  | rs72714876 | 15 | 37783271 | T | C | 0.385 | 0.084 | 4.57E-06 | 22.389 | 0.003 | 0.099 | 0.009 |
|  | rs9889896 | 17 | 32343860 | A | C | -0.142 | 0.031 | 3.47E-06 | 22.333 | 0.015 | 0.036 | 0.178 |
| Epilepsy (FinnGen) | rs10987903 | 9 | 130926865 | C | G | 0.1519 | 0.0331 | 4.37E-06 | 24.5664 | 0.0050 | 0.0171 | 0.7694 |
|  | rs112183697 | 5 | 160443577 | G | A | -0.2449 | 0.0495 | 7.59E-07 | 25.0073 | 0.0542 | 0.0431 | 0.2088 |
|  | rs117117424 | 12 | 128785456 | C | T | 0.4755 | 0.1021 | 3.16E-06 | 22.1311 | -0.0409 | 0.0441 | 0.3541 |
|  | rs12660365 | 6 | 160809624 | A | G | 0.1569 | 0.0338 | 3.39E-06 | 22.0405 | 0.0160 | 0.0163 | 0.3269 |
|  | rs139468884 | 10 | 104581908 | A | G | -0.3777 | 0.0817 | 3.80E-06 | 21.9782 | 0.0787 | 0.1135 | 0.4880 |
|  | rs2063119 | 15 | 98329163 | A | T | 0.1195 | 0.0256 | 3.09E-06 | 22.5797 | -0.0042 | 0.0139 | 0.7648 |
|  | rs4558089 | 10 | 50309330 | C | T | 0.1574 | 0.0332 | 2.09E-06 | 22.9768 | -0.0167 | 0.0160 | 0.2989 |
|  | rs4952755 | 2 | 45447789 | G | C | -0.1293 | 0.0279 | 3.55E-06 | 22.7732 | -0.0010 | 0.0144 | 0.9450 |
|  | rs6676391 | 1 | 240869240 | T | A | 0.2808 | 0.0598 | 2.69E-06 | 23.4871 | -0.0430 | 0.0391 | 0.2720 |
|  | rs9889896 | 17 | 32343860 | A | C | -0.1420 | 0.0306 | 3.47E-06 | 22.3327 | -0.0006 | 0.0183 | 0.9733 |
| Epilepsy (UKB) | rs10987903 | 9 | 130926865 | C | G | 0.1519 | 0.0331 | 4.37E-06 | 24.5664 | -0.1063 | 0.0548 | 0.0261 |
|  | rs112183697 | 5 | 160443577 | G | A | -0.2449 | 0.0495 | 7.59E-07 | 25.0073 | 0.03261 | 0.0848 | 0.1547 |
|  | rs117117424 | 12 | 128785456 | C | T | 0.4755 | 0.1021 | 3.16E-06 | 22.1311 | 0.05203 | 0.1725 | 0.1175 |
|  | rs12660365 | 6 | 160809624 | A | G | 0.1569 | 0.0338 | 3.39E-06 | 22.0405 | 0.06249 | 0.0584 | 0.5457 |
|  | rs139468884 | 10 | 104581908 | A | G | -0.3777 | 0.0817 | 3.80E-06 | 21.9782 | 0.05499 | 0.1567 | 0.1393 |
|  | rs2063119 | 15 | 98329163 | A | T | 0.1195 | 0.0256 | 3.09E-06 | 22.5797 | -0.04749 | 0.0436 | 0.5587 |
|  | rs4558089 | 10 | 50309330 | C | T | 0.1574 | 0.0332 | 2.09E-06 | 22.9768 | -0.02241 | 0.0566 | 0.1597 |
|  | rs4952755 | 2 | 45447789 | G | C | -0.1293 | 0.0279 | 3.55E-06 | 22.7732 | -0.01178 | 0.0467 | 0.0965 |
|  | rs558293908 | 14 | 68995402 | G | T | -0.1548 | 0.0321 | 1.45E-06 | 27.8228 | 0.02107 | 0.0542 | 0.1565 |
|  | rs6676391 | 1 | 240869240 | T | A | 0.2808 | 0.0598 | 2.69E-06 | 23.4871 | -0.01273 | 0.0995 | 0.0467 |
|  | rs72714876 | 15 | 37783271 | T | C | 0.3846 | 0.0839 | 4.57E-06 | 22.3888 | -0.1657 | 0.1448 | 0.5982 |
|  | rs9889896 | 17 | 32343860 | A | C | -0.142 | 0.0306 | 3.47E-06 | 22.3327 | 0.01712 | 0.0520 | 0.1297 |
| Focal epilepsy (FinnGen) | rs10987903 | 9 | 130926865 | C | G | 0.1519 | 0.0331 | 4.37E-06 | 24.5664 | 0.0110 | 0.0232 | 0.6356 |
|  | rs112183697 | 5 | 160443577 | G | A | -0.2449 | 0.0495 | 7.59E-07 | 25.0073 | 0.0848 | 0.0584 | 0.1470 |
|  | rs117117424 | 12 | 128785456 | C | T | 0.4755 | 0.1021 | 3.16E-06 | 22.1311 | 0.0129 | 0.0594 | 0.8281 |
|  | rs12660365 | 6 | 160809624 | A | G | 0.1569 | 0.0338 | 3.39E-06 | 22.0405 | 0.0112 | 0.0221 | 0.6124 |
|  | rs139468884 | 10 | 104581908 | A | G | -0.3777 | 0.0817 | 3.80E-06 | 21.9782 | 0.0551 | 0.1551 | 0.7224 |
|  | rs2063119 | 15 | 98329163 | A | T | 0.1195 | 0.0256 | 3.09E-06 | 22.5797 | 0.0040 | 0.0189 | 0.8304 |
|  | rs4558089 | 10 | 50309330 | C | T | 0.1574 | 0.0332 | 2.09E-06 | 22.9768 | -0.0077 | 0.0217 | 0.7225 |
|  | rs4952755 | 2 | 45447789 | G | C | -0.1293 | 0.0279 | 3.55E-06 | 22.7732 | 0.0108 | 0.0196 | 0.5808 |
|  | rs6676391 | 1 | 240869240 | T | A | 0.2808 | 0.0598 | 2.69E-06 | 23.4871 | -0.0849 | 0.0533 | 0.1110 |
|  | rs9889896 | 17 | 32343860 | A | C | -0.142 | 0.0306 | 3.47E-06 | 22.3327 | -0.0387 | 0.0248 | 0.1187 |
| Focal epilepsy (UKB) | rs10987903 | 9 | 130926865 | C | G | 0.1519 | 0.0331 | 4.37E-06 | 24.5664 | -0.1022 | 0.0991 | 0.5198 |
|  | rs112183697 | 5 | 160443577 | G | A | -0.2449 | 0.0495 | 7.59E-07 | 25.0073 | 0.1025 | 0.1535 | 0.2975 |
|  | rs117117424 | 12 | 128785456 | C | T | 0.4755 | 0.1021 | 3.16E-06 | 22.1311 | -0.1507 | 0.3108 | 0.2022 |
|  | rs12660365 | 6 | 160809624 | A | G | 0.1569 | 0.0338 | 3.39E-06 | 22.0405 | 0.1210 | 0.1056 | 0.5988 |
|  | rs139468884 | 10 | 104581908 | A | G | -0.3777 | 0.0817 | 3.80E-06 | 21.9782 | 0.2578 | 0.2838 | 0.4393 |
|  | rs2063119 | 15 | 98329163 | A | T | 0.1195 | 0.0256 | 3.09E-06 | 22.5797 | -0.1079 | 0.0789 | 0.7657 |
|  | rs4558089 | 10 | 50309330 | C | T | 0.1574 | 0.0332 | 2.09E-06 | 22.9768 | -0.1430 | 0.1024 | 0.7896 |
|  | rs4952755 | 2 | 45447789 | G | C | -0.1293 | 0.0279 | 3.55E-06 | 22.7732 | 0.0130 | 0.0845 | 0.0565 |
|  | rs558293908 | 14 | 68995402 | G | T | -0.1548 | 0.0321 | 1.45E-06 | 27.8228 | -0.0524 | 0.0980 | 0.2270 |
|  | rs6676391 | 1 | 240869240 | T | A | 0.2808 | 0.0598 | 2.69E-06 | 23.4871 | -0.1647 | 0.1796 | 0.4447 |
|  | rs72714876 | 15 | 37783271 | T | C | 0.3846 | 0.0839 | 4.57E-06 | 22.3888 | -0.0485 | 0.2624 | 0.0688 |
|  | rs9889896 | 17 | 32343860 | A | C | -0.142 | 0.0306 | 3.47E-06 | 22.3327 | 0.0544 | 0.0940 | 0.2498 |

Abbreviations: BDNF, brain-derived neurotrophic factor; SNP, single-nucleotide polymorphism; Chr, chromosome; Pos, position; SE, standard error; UKB, UK Biobank.

**Supplementary Figures**

**Fig. S1.** Plasma BDNF and stroke risk. (A) Scatter plot, (B) Funnel plot, (C) Forest plot, and (D) Leave-one-out plot. BDNF, brain-derived neurotrophic factor.

**Fig. S2.** Plasma BDNF and ischemic stroke risk. (A) Scatter plot, (B) Funnel plot, (C) Forest plot, and (D) Leave-one-out plot. BDNF, brain-derived neurotrophic factor.

**Fig. S3.** Plasma BDNF and ischemic stroke (large artery atherosclerosis) risk. (A) Scatter plot, (B) Funnel plot, (C) Forest plot, and (D) Leave-one-out plot. BDNF, brain-derived neurotrophic factor.

**Fig. S4.** Plasma BDNF and ischemic stroke (small-vessel) risk. (A) Scatter plot, (B) Funnel plot, (C) Forest plot, and (D) Leave-one-out plot. BDNF, brain-derived neurotrophic factor.

**Fig. S5.** Plasma BDNF and ischemic stroke (cardioembolic) risk. (A) Scatter plot, (B) Funnel plot, (C) Forest plot, and (D) Leave-one-out plot. BDNF, brain-derived neurotrophic factor.

**Fig. S6.** Plasma BDNF and AD risk. (A) Scatter plot, (B) Funnel plot, (C) Forest plot, and (D) Leave-one-out plot. AD, Alzheimer’s disease; BDNF, brain-derived neurotrophic factor.

**Fig. S7.** Plasma BDNF and AD (early onset) risk. (A) Scatter plot, (B) Funnel plot, (C) Forest plot, and (D) Leave-one-out plot. AD, Alzheimer’s disease; BDNF, brain-derived neurotrophic factor.

**Fig. S8.** Plasma BDNF and AD (late onset) risk. (A) Scatter plot, (B) Funnel plot, (C) Forest plot, and (D) Leave-one-out plot. AD, Alzheimer’s disease; BDNF, brain-derived neurotrophic factor.

**Fig. S9.** Plasma BDNF and PD risk. (A) Scatter plot, (B) Funnel plot, (C) Forest plot, and (D) Leave-one-out plot. PD, Parkinson’s disease; BDNF, brain-derived neurotrophic factor.

**Fig. S10.** Plasma BDNF and ALS risk. (A) Scatter plot, (B) Funnel plot, (C) Forest plot, and (D) Leave-one-out plot. ALS, amyotrophic lateral sclerosis; BDNF, brain-derived neurotrophic factor.

**Fig. S11.** Plasma BDNF and MS risk. (A) Scatter plot, (B) Funnel plot, (C) Forest plot, and (D) Leave-one-out plot. MS, multiple sclerosis; BDNF, brain-derived neurotrophic factor.

**Fig. S12.** Plasma BDNF and focal epilepsy-documented hippocampal sclerosis risk. (A) Scatter plot, (B) Funnel plot, (C) Forest plot, and (D) Leave-one-out plot. BDNF, brain-derived neurotrophic factor.

**Fig. S13.** Plasma BDNF and focal epilepsy-documented lesion other than hippocampal sclerosis risk. (A) Scatter plot, (B) Funnel plot, (C) Forest plot, and (D) Leave-one-out plot. BDNF, brain-derived neurotrophic factor.

**Fig. S14.** Plasma BDNF and generalized epilepsy risk. (A) Scatter plot, (B) Funnel plot, (C) Forest plot, and (D) Leave-one-out plot. BDNF, brain-derived neurotrophic factor.

**Fig. S15.** Plasma BDNF and juvenile absence epilepsy risk. (A) Scatter plot, (B) Funnel plot, (C) Forest plot, and (D) Leave-one-out plot. BDNF, brain-derived neurotrophic factor.

**Fig. S16.** Plasma BDNF and childhood absence epilepsy risk. (A) Scatter plot, (B) Funnel plot, (C) Forest plot, and (D) Leave-one-out plot. BDNF, brain-derived neurotrophic factor.

**Fig. S17.** Plasma BDNF and generalized epilepsy with tonic-clonic seizures risk. (A) Scatter plot, (B) Funnel plot, (C) Forest plot, and (D) Leave-one-out plot. BDNF, brain-derived neurotrophic factor.

**Fig. S18.** Plasma BDNF and juvenile myoclonic epilepsy risk. (A) Scatter plot, (B) Funnel plot, (C) Forest plot, and (D) Leave-one-out plot. BDNF, brain-derived neurotrophic factor.

**Fig. S19.** Plasma BDNF and migraine risk. (A) Scatter plot, (B) Funnel plot, (C) Forest plot, and (D) Leave-one-out plot. BDNF, brain-derived neurotrophic factor.

**Fig. S20.** Plasma BDNF and migraine (with aura) risk. (A) Scatter plot, (B) Funnel plot, (C) Forest plot, and (D) Leave-one-out plot. BDNF, brain-derived neurotrophic factor.

**Fig. S21.** Plasma BDNF and migraine (without aura, drug-induced) risk. (A) Scatter plot, (B) Funnel plot, (C) Forest plot, and (D) Leave-one-out plot. BDNF, brain-derived neurotrophic factor.

**Fig. S22.** Plasma BDNF and migraine (without aura and triptan purchases) risk. (A) Scatter plot, (B) Funnel plot, (C) Forest plot, and (D) Leave-one-out plot. BDNF, brain-derived neurotrophic factor.

**Fig. S23.** Plasma BDNF and intracranial hemorrhage (UKB) risk. (A) Scatter plot, (B) Funnel plot, (C) Forest plot, and (D) Leave-one-out plot. UKB, UK Biobank. BDNF, brain-derived neurotrophic factor.

**Fig. S24.** Plasma BDNF and epilepsy (FinnGen) risk. (A) Scatter plot, (B) Funnel plot, (C) Forest plot, and (D) Leave-one-out plot. BDNF, brain-derived neurotrophic factor.

**Fig. S25.** Plasma BDNF and focal epilepsy (FinnGen) risk. (A) Scatter plot, (B) Funnel plot, (C) Forest plot, and (D) Leave-one-out plot. BDNF, brain-derived neurotrophic factor.

**Fig. S26.** Plasma BDNF and epilepsy (UKB) risk. (A) Scatter plot, (B) Funnel plot, (C) Forest plot, and (D) Leave-one-out plot. UKB, UK Biobank. BDNF, brain-derived neurotrophic factor.

**Fig. S27.** Plasma BDNF and focal epilepsy (UKB) risk. (A) Scatter plot, (B) Funnel plot, (C) Forest plot, and (D) Leave-one-out plot. UKB, UK Biobank. BDNF, brain-derived neurotrophic factor.
